# A candidate subunit vaccine induces protective immunity against *Mycobacterium avium* subspecies *paratuberculosis* in mice

**DOI:** 10.1038/s41541-023-00675-1

**Published:** 2023-05-20

**Authors:** Mingzhu Shao, Ning Cui, Yangyang Tang, Fanruo Chen, Yingying Cui, Guanghui Dang, Siguo Liu

**Affiliations:** State Key Laboratory for Animal Disease Control and Prevention, Division of Bacterial Diseases, Harbin Veterinary Research Institute, Chinese Academy of Agricultural Sciences, 678 Haping Street, Harbin, 150069 PR China

**Keywords:** Bacteriology, Protein vaccines

## Abstract

*Mycobacterium avium* subspecies *paratuberculosis* (MAP) causes paratuberculosis (PTB), which is a granulomatous enteritis in ruminants that threatens the dairy industry’s healthy development and public health safety worldwide. Because the commercial inactivated vaccines are not completely protective and interfere with bovine tuberculosis diagnostics, we tested four fusion proteins, namely 66NC, 66CN, 90NC, and 90CN, which were constructed with MAP3527, Ag85B, and Hsp70 of MAP in different tandem combinations. Notably, 66NC, which encodes a 66 kDa fusion protein that combines in linear order MAP3527_N40–232_, Ag85B_41–330_, and MAP3527_C231–361,_ induced a powerful and specific IFN-γ response. Immunization of C57BL/6 mice with the 66NC fusion protein formulated in Montanide ISA 61 VG adjuvant generated robust Th1, Th2, and Th17 type immune responses and strong antibody responses. The 66NC vaccine protected C57BL/6 mice against virulent MAP K-10 infection. This resulted in a reduction of bacterial load and improvement of pathological damage in the liver and intestine, in addition to a reduction of body weight loss; significantly better protection than the reported 74 F vaccine was also induced. Furthermore, vaccine efficacy correlated with the levels of IFN-γ-, TNF-α-, and IL-17A-secreting antigen-specific CD4^+^ and CD8^+^ T lymphocytes as well as with serum IFN-γ and TNF-α levels after vaccination. These results demonstrate that recombinant protein 66NC is an efficient candidate for further development into a protective vaccine in terms of inducing specific protection against MAP.

## Introduction

*Mycobacterium avium* subspecies *paratuberculosis* (MAP) causes paratuberculosis (PTB), commonly referred to as Johne’s disease, which is a long-term granulomatous inflammation of the intestines of both domestic and wild ruminants. Weight loss, severe diarrhea, and a decrease in milk yield and body condition are among the clinical signs associated with this condition as it progresses^1^. PTB shows a worldwide distribution and a high incidence, and it threatens the sustainable and healthy development of the dairy industry^2,3^. It is estimated that a majority of US dairy herds, approximately 90%, are infected with MAP^4^, leading to an annual economic loss of around 250 million dollars in the United States^3^. In addition, MAP has been detected in powdered infant formula and retail pasteurized milk^5,6^, and the pathogen has been found in numerous human diseases such as Crohn’s disease, type I diabetes, and Hashimoto’s thyroiditis^7–9^, threatening global public health and socioeconomic security.

Despite the severity of PTB in ruminants, no effective vaccine has been developed. Three previously used vaccines (Mycopar, Silirum, and Gudair) are all inactivated whole-cell MAP formulated in oil emulsions. However, these vaccines are not completely protective^1^ and cause local granulomas and abscess formation at the injection site, the likely main obstacle being the interference with a diagnostic test regularly employed for bovine tuberculosis and PTB^1,10^. For any effective PTB control program, the most important factor is reducing the fecal shedding of MAP. However, inactivated vaccines have been found only to bring about a minimal reduction in fecal shedding^11,12^. Therefore, researchers in the United States worked together through the Johne’s Disease Integrated Program and used a gene knockout approach to develop modified live vaccines, which appear to stop the fecal shedding of MAP^1,10^. Indeed, live attenuated MAP vaccines provide better protection but potential interference in tuberculosis diagnostic tests is not eliminated. Moreover, the possibility of inadvertent human exposure to modified live MAP is also a considerable public health concern^13,14^.

Therefore, there is an urgent need for innovative and effective vaccines against PTB that do not interfere with bovine TB diagnostics. To address these issues, researchers are exploring a range of solutions, such as subunit vaccines and DNA-based vaccines. MAP heat shock protein 70 (Hsp70) is an immunodominant antigen^15^, and the recombinant MAP Hsp70 vaccination reduces the shedding of bacteria in feces for a long period in calves^16^. A study has revealed that the Hsp70 vaccine does not impede the recognition of tuberculosis, and animals vaccinated with Hsp70 are easily distinguishable from those infected with MAP^17^. Cattle and mice infected with MAP induce a rapid and strong T-cell response against culture filtrate proteins generally and Ag85B proteins in particular^18^. Recombinant MAP Ag85B emulsified in Ribi adjuvant induced both Th1- and Th2-type immune response^19^. The Ag85B antigen from MAP was expressed in transgenic alfalfa and elicited long-lasting immunity in mice^20^. A fusion protein 74 F consisting of MAP3527 and MAP1519 admixed with the MPL adjuvant and another fusion protein particle vaccine containing MAP antigens Ag85A-SOD-Ag85B-74F effectively protected mice against MAP infection^21,22^. Moreover, an attenuated strain of Salmonella carrying the SopE104-Ag85A-SOD-Ag85B and SopE104-74F genes elicited a robust and persistent Th1 immune response characterized by production of IFN-γ, providing protection in mice against MAP challenge^23^. To develop subunit vaccines against PTB, it is important to identify mycobacterial antigens that preferentially activate CD4^+^ and CD8^+^ T cells to proliferate and secrete IFN-γ^1,21,24^. Previous studies have identified three MAP antigens (MAP3527, Ag85B, and Hsp70) in the conditions of controlled infection in sheep, bovine, and mice characterized by their capability to induce T cell and antibody responses^16,18,21^. In this study, we constructed four fusion proteins by tandemly combining MAP3527, Ag85B, and Hsp70 in different combinations, named 66NC, 66CN, 90NC, and 90CN, according to their molecular weight. We further investigated the immunoprotective properties of these fusion proteins in a mouse model.

Notably, ruminant infection models are widely used for PTB vaccination studies^24,25^. However, due to the lengthy incubation of the disease in ruminants and the expense of conducting experiments, non-ruminant laboratory animals have been developed as a substitute for ruminants. C57BL/6 mice are frequently utilized as models to investigate MAP infections and vaccine development^21,26,27^. This study focuses on the immunogenicity and protective efficacy of the fusion protein 66NC formulated with the Montanide ISA 61 VG adjuvant, which was studied and compared with the polyprotein 74 F in a murine model.

## Results

### Generation and purification of recombinant 66NC, 66CN, 90NC, and 90CN

**Fig. 1:**
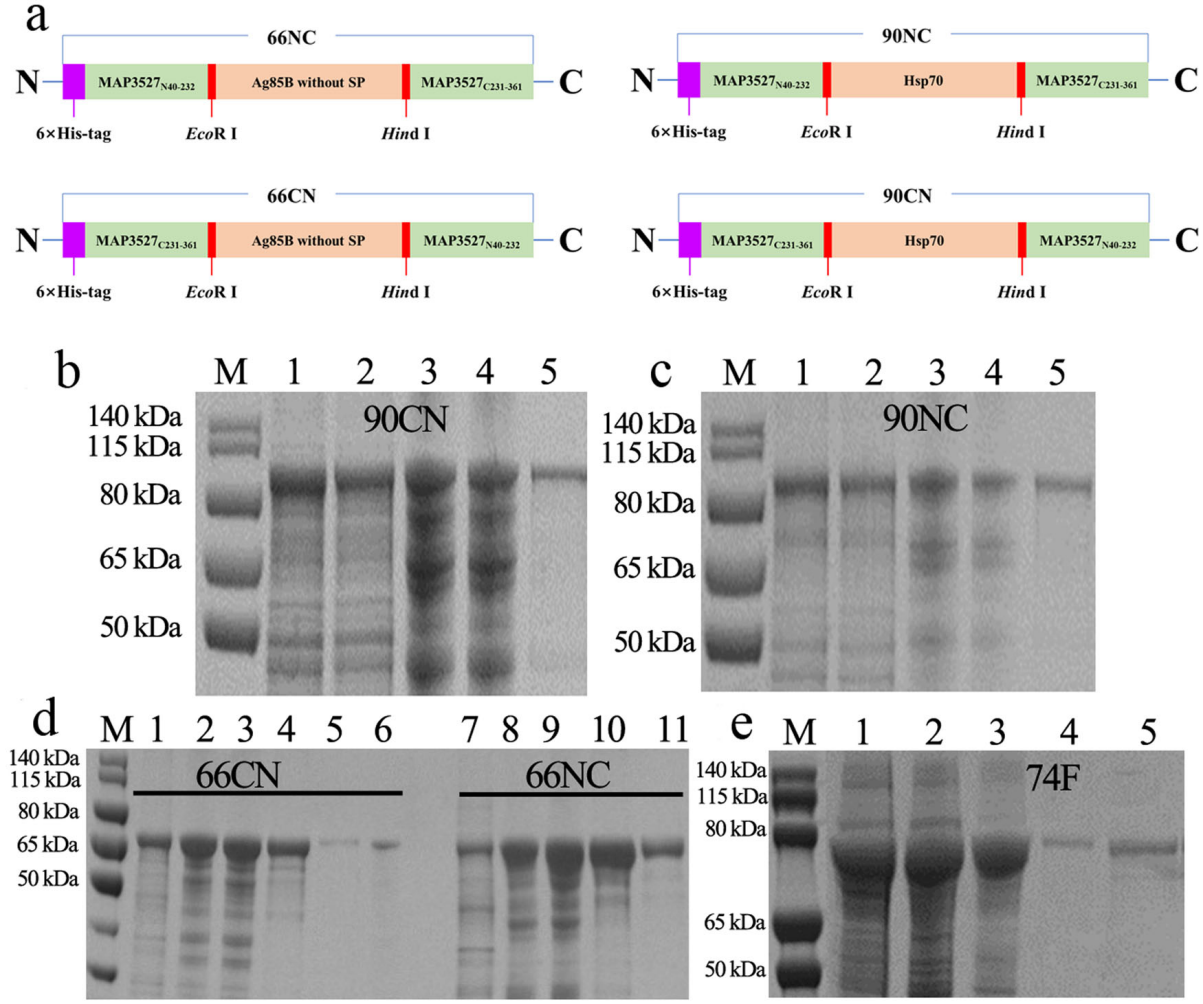
Generation and purification of recombinant fusion proteins. **a** Schematic representation of 66NC, 66CN, 90NC, and 90CN constructs. **b** Purification of a recombinant 90CN fusion protein. line M, protein molecular weight marker; line 1, 90CN protein after renaturation; line 2, flow-through from the resin; line 3, protein-bound resin before washing; line 4, protein-bound resin after washing; line 5, purified 90CN protein eluted by 500 mM imidazole. **c** Purification of a recombinant 90NC fusion protein. line M, protein molecular weight marker; line 1, 90NC protein after renaturation; line 2, flow-through from the resin; line 3, protein-bound resin before washing; line 4, protein-bound resin after washing; line 5, purified 90NC protein eluted by 500 mM imidazole. **d** Purification of the recombinant 66CN and 66NC fusion protein. line M, protein molecular weight marker; line 1, flow-through from the resin; line 2, protein-bound resin before washing; line 3, protein-bound resin after washing; line 4 and 5, purified 66CN protein eluted by 500 mM and 1 M imidazole, respectively; line 6, protein-bound resin after elution; line 7, flow-through from the resin; line 8, protein-bound resin before washing; line 9, protein-bound resin after washing; line 10 and 11, purified 66NC protein eluted by 500 mM and 1 M imidazole, respectively. **e** Purification of a recombinant 74 F fusion protein. line M, protein molecular weight marker; line 1, flow-through from the resin; line 2, protein-bound resin before washing; line 3, protein-bound resin after washing; line 4 and 5, purified 74 F protein eluted by 200 mM and 500 mM imidazole, respectively. All gels derive from the same experiment and they were processed in parallel.

A diagrammatic representation of 66NC, 66CN, 90NC, and 90CN showing the organization and restriction enzyme sites (*Eco*R I and *Hin*d III) used to construct the fusion protein and data on the protein purification using an N-terminus six-histidine-tag are shown in Fig. 1a. The nucleotide and amino acid sequences of four fusion proteins are available in Supplementary Sequence 1. The ORF lengths of 66NC, 66CN, 90NC, and 90CN are 1902 bp, 1902 bp, 2547 bp, and 2547 bp, encoding 653 aa, 653 aa, 868 aa, and 838 aa, respectively, with predicted molecular masses of ~66 kDa, ~66 kDa, ~90 kDa, and ~90 kDa, respectively. The expressed recombinant protein in the *E. coil* was purified by Ni-column affinity chromatography (Fig. 1b–d). The purification of polyprotein 74 F was accomplished following previous reports^21^ and analyzed by SDS-PAGE (Fig. 1e).

### Screening of the adjuvants, immunization routes, and immunogens

**Fig. 2:**
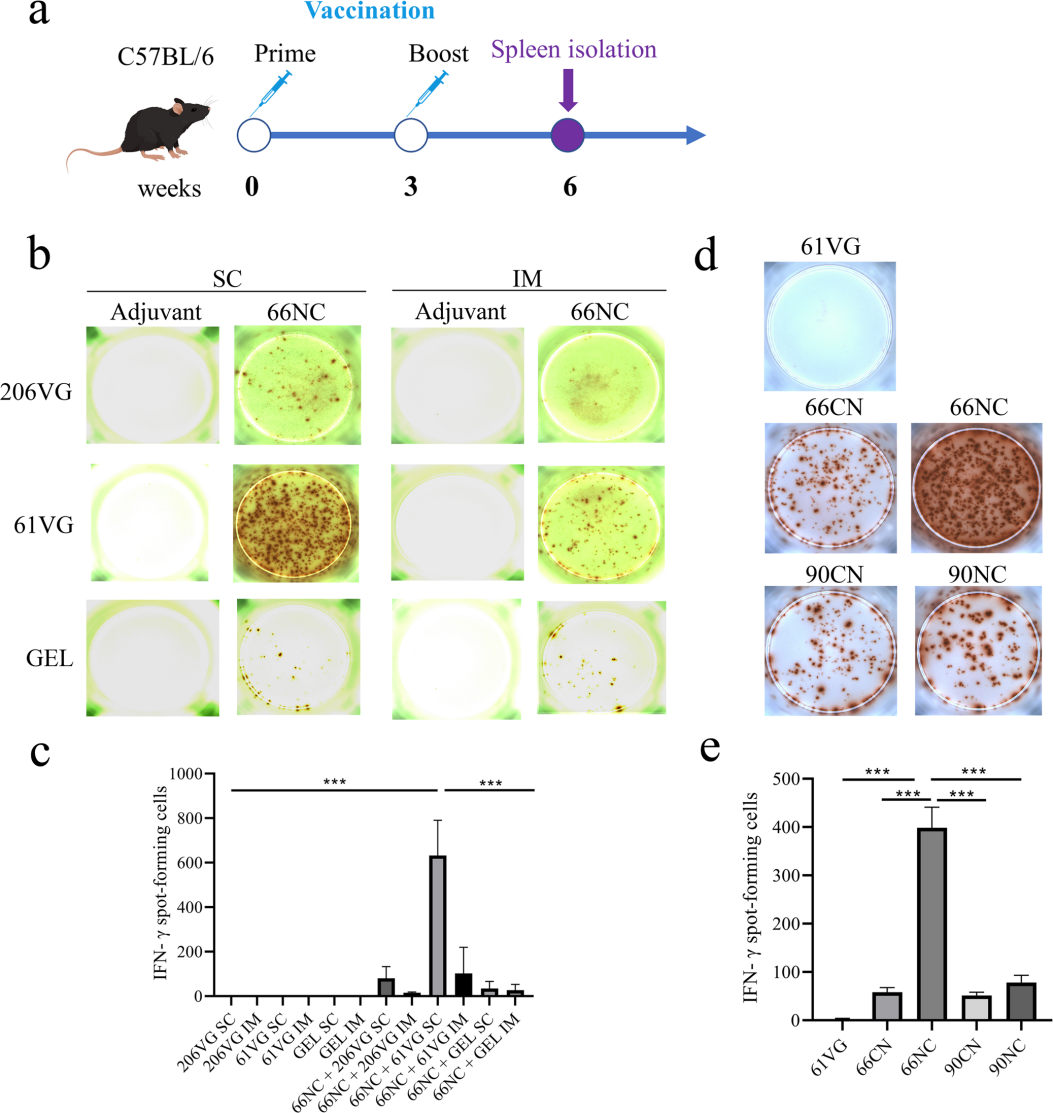
Screening of adjuvants, injection routes, and immunogens through an IFN-γ ELISpot assay. **a** Schematic diagram of the vaccination timeline. **b**, **c** The IFN-γ ELISpot assay was used to determine antigen-specific IFN-γ expression in splenic lymphocytes of mice immunized intramuscularly (IM) and subcutaneously (SC) with 66NC fusion protein formulated in different adjuvants Montanide ISA 206 VG (206 VG), Montanide ISA 61 VG (61 VG), and Montanide GEL 02 PR (GEL) and stimulated with the indicated antigen, with the adjuvant alone as a negative control. **b** Representative pictures of the IFN-γ ELISpot assay (representative experiment with at least three independent replicates). **c** Quantitation of the IFN-γ ELISpot assay. **d**, **e** The IFN-γ ELISpot assay was used to determine antigen-specific IFN-γ expression in splenic lymphocytes of mice immunized subcutaneously (SC) with fusion protein (66NC, 66NC, 90NC, and 90CN) formulated in Montanide ISA 61 VG adjuvant and stimulated with the indicated antigen, with the adjuvant alone as a negative control. **d** Pictures of the IFN-γ ELISpot assay (representative of one experiment with at least three independent replicates). **e** Quantitation of the IFN-γ ELISpot assay. **c**, **e** Error bars indicate the Standard Error of Mean (SEM). One-way ANOVA was used to analyze statistical significance, followed by Tukey’s multiple comparisons tests. ***P < 0.001.

IFN-γ is critical in combating intracellular pathogens, such as mycobacteria^28,29^. To screen the optimal adjuvants and immunization routes, we immunized mice subcutaneously or intramuscularly with 66NC fusion protein emulsified with Montanide ISA 61 VG, Montanide GEL 02 PR, or Montanide ISA 206 VG. Three weeks following the second immunization, splenocytes from the immunized mice were collected for IFN-γ ELISpot assay (Fig. 2a). Subcutaneous immunization of mice with 66NC formulated in Montanide ISA 61 VG adjuvant resulted in the production of the strongest IFN-γ response after in vitro stimulation of splenic lymphocytes co-cultures with 66NC fusion protein compared to those of the other 11 groups (Fig. 2b, c). To screen the optimal immunogens, we immunized mice subcutaneously with Montanide ISA 61 VG adjuvant mixed with 66NC, 66CN, 90NC, or 90CN fusion protein. Immunization of mice with 66NC stimulated the most robust production of IFN-γ reaction against 66NC fusion protein compared to 66CN, 90NC, and 90CN immunized mice (Fig. 2d, e). The above results demonstrate that the best route of immunization, adjuvant, and immunogen was a subcutaneous injection, Montanide ISA 61 VG adjuvant, and 66NC fusion protein, respectively, which induced a strong antigen-specific IFN-γ response in C57BL/6 mice.

### Immune responses to 66NC fusion protein formulated in 61VG adjuvant

**Fig. 3:**
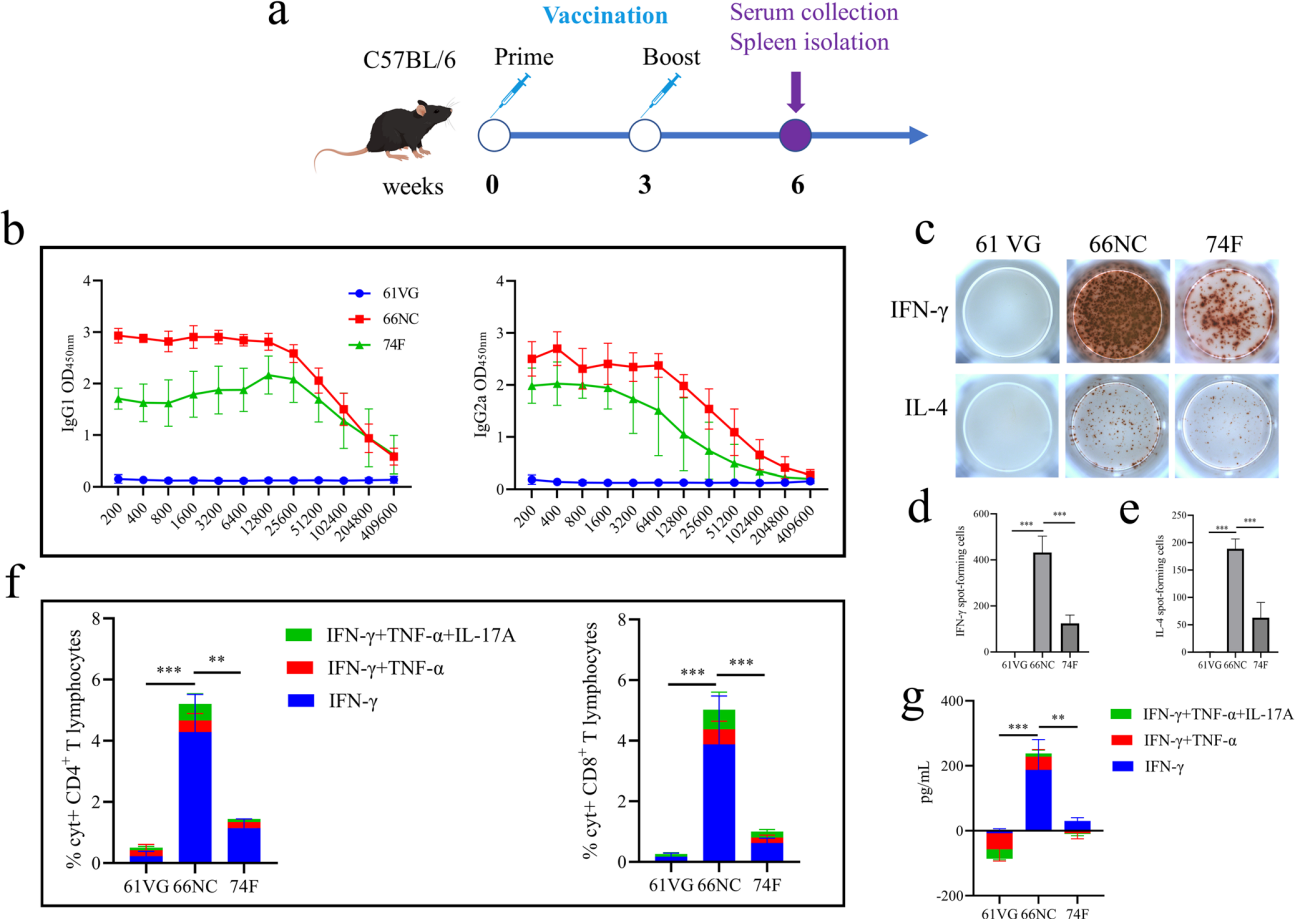
Immune responses in C57BL/6 mice immunized with the 66NC fusion protein in Montanide ISA 61 VG adjuvant. **a** Schematic diagram of the vaccination timeline. C57BL/6 mice were subcutaneously injected two times (three weeks apart) with 50 μg 66NC fusion protein formulated in Montanide ISA 61 VG adjuvant. Control groups include mice immunized with Montanide ISA 61 VG adjuvant alone as a negative control or 74 F mixed MPL adjuvant as subunit vaccine control. Three weeks after the booster immunization, the mice were bled and sacrificed. **b** Antigen-specific IgG1 and IgG2a isotypes were measured by ELISA. **c** Pictures of the IFN-γ and IL-4 ELISpot assay (representative of one experiment with at least five independent replicates). **d**, **e** Quantitation of the IFN-γ and IL-4 ELISpot assay. **f** The percentage of cytokine-positive (%Cyt^+^) antigen-specific T-lymphocytes was quantified by intracellular cytokine staining (ICS) in CD4^+^ and CD8^+^ following stimulation of splenic T lymphocytes with the indicated antigen. **g** Serum cytokines levels of IFN-γ, TNF-α, and IL-17A were measured by ELISA. **d**–**g** Error bars indicate the Standard Error of Mean (SEM). One-way ANOVA was used to analyze the statistical significance, followed by Tukey’s multiple comparisons tests. **P < 0.01, ***P < 0.001.

Mice were immunized two times, at two-week intervals, with 50 μg of 66NC mixed in 61VG adjuvant via the subcutaneous route, and 74 F polyprotein was used as a subunit vaccine control. Three weeks post the second immunization, six mice from each group were sacrificed, and anti-66NC antibody, cytokines secretion, and T cell response were assessed (Fig. 3a). With respect to antibody responses, mice immunized with 66NC showed robust IgG1 and IgG2a responses against 66NC fusion protein and the antibody levels of the mice immunized with 66NC were significantly higher than those of the mice immunized with 74 F (Fig. 3b). The adjuvant-alone control groups did not exhibit any specific responses to 66NC.

Th1 cells produce cytokines such as IFN-γ, TNF-α, and IL-2, with IFN-γ being the cytokine that characterizes the lineage^30^. Compared to Th1 cells, Th2 cells generate IL-4, IL-5, and IL-13, and IL-4 is the primary cytokine that contributes to the transformation of naive T cells into Th2 cells^30^. Th1- and Th2-immune responses were evaluated by IFN-γ and IL-4 ELISpot assay. 66NC vaccination induced higher relative numbers of IFN-γ- and IL-4-expressing splenic lymphocytes than 74 F vaccination or only adjuvant vaccination (Fig. 3c–e).

Subsequently, we evaluated the antigen-specific T-lymphocyte reactions in the spleen through ICS analyses. Higher IFN-γ-, TNF-α-, and IL-17A-secreting CD4^+^ T and CD8^+^ T cell responses were observed in the spleen of 66NC vaccinated mice as compared to 74 F vaccinated mice (Fig. 3f). Additionally, levels of IFN-γ, TNF-α, and IL-17A in sera were determined by ELISA. The levels of these three cytokines were higher in mice immunized with 66NC compared to mice immunized with 74 F or given only adjuvant (Fig. 3g). These data suggest that 66NC vaccinated mice displayed Th1, Th2, and Th17 type immune responses and strong antibody responses.

### 66NC vaccine protects C57BL/6 mice against MAP K-10 infection and induced significantly better protection than the 74 F vaccine

**Fig. 4:**
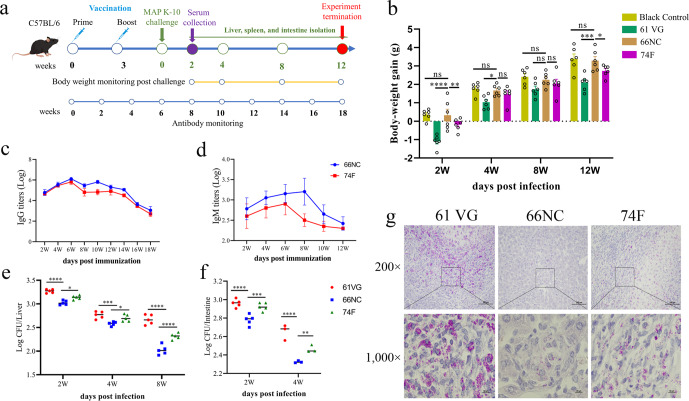
66NC protects C57BL/6 mice against intraperitoneal challenge with MAP K-10. **a** Schematic diagram of the vaccination and MAP challenge timeline. C57BL/6 mice were subcutaneously injected two times (three weeks apart) with 50 μg 66NC fusion protein formulated in Montanide ISA 61 VG (61 VG) adjuvant. Control groups include mice immunized with Montanide ISA 61 VG adjuvant alone as a negative control or 74 F mixed MPL adjuvant as subunit vaccine control. Three weeks after the booster immunization, the mice were challenged with MAP K-10 by intraperitoneal injection. **b** Body weight was measured for 2, 4, 8, and 12 weeks after the MAP challenge (*n* = 6 per group). Monitor IgG (**c**) and IgM (**d**) antibody levels. Sera were collected and measured for specific anti-66NC or 74 F antibody response of both IgG and IgM at 2, 4, 6, 8, 10, 12, 14, 16, and 18 weeks after prime vaccination by ELISA. Bacterial load quantification was evaluated in the liver (**e**) and intestine (**f**) from mice challenged by intraperitoneal injection with MAP K-10 for 2, 4, and 8 weeks after vaccination. **g** Representative images of Ziehl-Neelsen acid-fast stain in the liver of vaccinated mice, two weeks after being challenged with MAP, are presented (scale bars, 100 μm (top) and 10 μm (bottom)). The bottom images are enlarged from the outlined areas of the top images. **b**, **e**, **f** Data indicate cumulative results from at least three to six independent replicates. Mean ± SEM and Two-way ANOVA were used to analyze the statistical significance, followed by Tukey’s multiple comparisons tests. ns, non-significant; *P < 0.05; **P < 0.01; ***P < 0.001; ****P < 0.0001.

At week six, vaccinated mice were challenged by the intraperitoneal route with 10^9^ CFU of MAP K-10, and euthanized at weeks 2, 4, 8, and 12 following the challenge to evaluate protective efficacy (Fig. 4a). In the clinical stage of PTB, symptoms include persistent diarrhea, weight loss, and progressive emaciation^31^. During the experimental period, we monitored the body weight of the vaccinated mice at 2, 4, 8, and 12 weeks post-challenge. Weight gain of 66NC vaccinated mice was comparable to that of blank mice and significantly higher than in the 74 F or adjuvant-alone vaccinated mice at week 2 following the challenge. These differences persisted at weeks 4, 8, and 12 (Fig. 4b). It is suggested that humoral immunity could be beneficial in the fight against MAP^32^. The antibody levels in sera at weeks 2–18, measured at 2-week intervals after immunization were determined by ELISA. The levels of the IgG and IgM of the mice immunized with 66NC were higher than those of the mice immunized with 74 F or adjuvant alone, and the IgM antibody was sustained up to 12 weeks post-vaccination (Fig. 4c, d). Remarkably, the IgG antibody titer remained at a high level until 18 weeks (Fig. 4c). Bacterial burden (CFU) was measured in the liver and intestine of mice at different time points post-challenge. In the liver, MAP load was significantly decreased in the 66NC vaccinated mice compared to the 74 F or adjuvant-alone vaccinated mice at 2, 4, and 8 weeks after the challenge (Fig. 4e). Qualitatively similar results were observed in the liver of vaccinated mice at two weeks post-challenge using Ziehl-Neilsen staining (Fig. 4g). Mice vaccinated with 66NC showed significantly reduced MAP loads in the intestine compared to 74 F or adjuvant-alone vaccinated mice at 2 and 4 weeks after the MAP K-10 challenge (Fig. 4f). No bacteria were cultured from the liver at 12 weeks and the intestine at eight weeks and 12 weeks post-challenge.

**Fig. 5:**
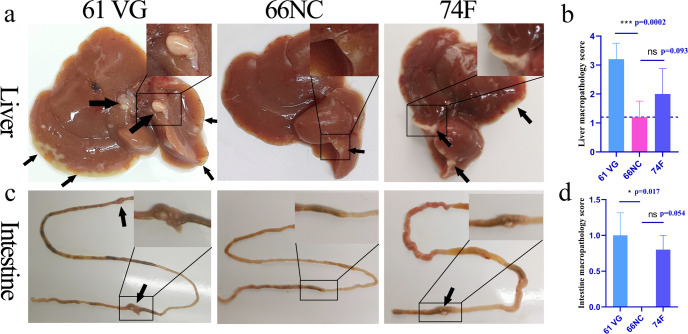
Macropathological findings of the liver and intestine. To evaluate macropathology, the liver and intestine of mice challenged by intraperitoneal injection with MAP K-10 for two weeks after vaccination were photographed. An image represents one experiment with at least five independent replicates. **a** Macropathological changes in the liver. Montanide ISA 61 VG (61 VG): numerous macro-granulomas (black arrow), 66NC: few micro-granulomas (black arrow), 74 F: numerous macro-granulomas (black arrow). **b** Liver macropathology score. **c** Macropathological changes in the intestine. Montanide ISA 61 VG (61 VG): macro-granulomas (black arrow), 66NC: no visible pathology, 74 F: macro-granulomas (black arrow). **d** Intestine macropathology score. **b**, **d** mean ± SEM, and One-way ANOVA were used to analyze the statistical significance, followed by Tukey’s multiple comparisons tests. ns, non-significant; *P < 0.05; ***P < 0.001.

Macropathology and histopathology was conducted on liver and intestines samples obtained after necropsy of mice at 2 weeks post-challenge. Numerous granulomas on the liver surface were seen in mice immunized with 74 F and adjuvant only. In contrast, the granulomas were fewer and smaller in mice immunized with 66NC (Fig. 5a, b, black arrow). In addition, visible macro-granulomas on the intestine were observed in mice immunized with 74 F and adjuvant only, while 66NC vaccinated mice had no visible pathology (Fig. 5c, d, black arrow). Regarding histopathology, the liver samples from mice given adjuvant alone showed multiple micro-granuloma (black arrow), and focal visible large areas of necrosis resulted in the formation of macro-granulomas (green arrow).

**Fig. 6:**
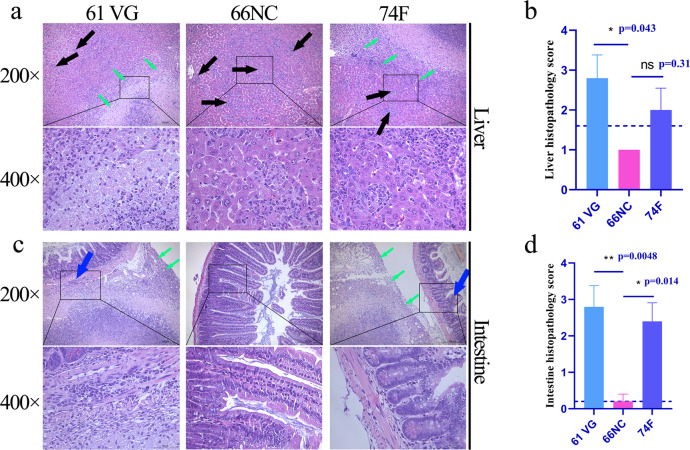
Histopathological findings of the liver and intestine. Histopathology was evaluated in the liver and intestine sections stained with hematoxylin and eosin from mice challenged by intraperitoneal injection with MAP K-10 for 2 weeks after vaccination. An image represents one experiment with at least five independent replicates. **a** Histopathological changes in the liver (scale bars, 200 μm (top) and 50 μm (bottom)). Montanide ISA 61 VG (61 VG): macro-granulomas (green arrow) and multiple microgranulomas (black arrow), 66NC: multiple microgranulomas (black arrow), 74 F: macro-granulomas (green arrow) and multiple microgranulomas (black arrow). The bottom images are enlarged from the outlined areas of the top images. **b** Liver histopathology score. **c** Histopathological changes in the intestine (scale bars, 200 μm (top) and 50 μm (bottom)). Montanide ISA 61 VG (61 VG): macro-granulomas (green arrow) and inflammatory cell infiltration (blue arrow), 66NC: without pathologies, 74 F: macro-granulomas (green arrow) and inflammatory cell infiltration (blue arrow). The bottom images are enlarged from the outlined areas of the top images. **d** Intestine histopathology score. **b**, **d** Mean ± SEM and one-way ANOVA were used to analyze statistical significance followed by Tukey’s multiple comparisons tests. ns, non-significant; *P < 0.05; **P < 0.01.

In contrast, the liver of the 66NC vaccinated mice had only multiple microgranulomas (black arrow; Fig. 6a, b). In addition, visible macro-granulomas of serosa (green arrow) and multiple microgranulomas in the parenchyma (black arrow) were seen in the 74 F vaccinated mice. Likewise, intestines of only adjuvant or 74 F vaccinated mice had a macro-granuloma on the serosal surface (green arrow). In addition, many inflammatory cell infiltrates in muscularis or lamina propria (blue arrow). In contrast, the intestine of the 66NC vaccinated mice was without pathologies (Fig. 6c, d). The results above suggest that the 66NC fusion protein formulated in Montanide ISA 61 VG adjuvant contributed to the improved protection of C57BL/6 mice against MAP K-10 and is better than the 74 F fusion protein mixed in MPL adjuvant.

### 66NC vaccination is associated with increased expression of IFN-γ, TNF-α, and IL-17A in antigen-specific T cells or serum and correlates with accelerated bacterial clearance after the MAP K-10 challenge

**Fig. 7:**
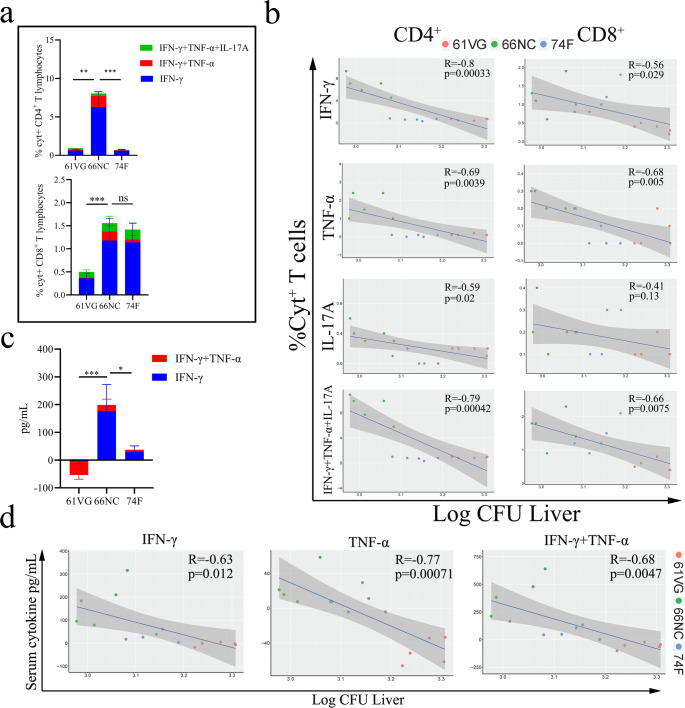
Cellular immune responses post MAP challenge of vaccinated C57BL/6 mice. **a** Percentage of cytokine-positive (Cyt^+^) splenic CD4+ and CD8 + T cells measured by intracellular cytokine staining two weeks post MAP challenge of mice vaccinated with the indicated antigen. **b** Pearson’s correlation (R) of Cyt^+^ CD4^+^ and CD8^+^ T lymphocytes with CFU in the liver following challenge. **c** Serum cytokine levels from Montanide ISA 61 VG (61 VG) adjuvant and vaccinated mice (66NC and 74 F) were assessed 2 weeks after being challenged by MAP K-10. **d** Pearson’s correlations of cytokine levels with CFU in the liver following challenge for IFN-γ and TNF-α. Data representative of one experiment with at least five independent replicates, mean ± SEM. One-way ANOVA was used to analyze the statistical significance, followed by Tukey’s multiple comparisons tests. ns, non-significant; *P < 0.05; **P < 0.01; ***P < 0.001.

To investigate the correlation between cytokine levels and bacterial load (CFU) in mice after immunization, we evaluated the antigen-specific cytokine-secreting (Cyt + ), CD4^+^, or CD8^+^ T lymphocytes from spleens and the level of serum cytokines of vaccinated mice at two weeks following the MAP K-10 challenges. 66NC immunization resulted in higher IFN-γ-, TNF-α-, and IL-17A-secreting antigen-specific CD4^+^ or CD8^+^ T cell responses in the spleen than did 74 F vaccination or adjuvant-alone after challenge (Fig. 7a), and consistent results were observed for the level of IFN-γ and TNF-α in serum of immunized mice against MAP K-10; however, unfortunately, IL-17A was not detected in serum (Fig. 7c). Among IFN-γ- (*P* = 0.00033 or *P* = 0.029), TNF-α- (*P* = 0.0039 or *P* = 0.005), IL-17A- (*P* = 0.02 or *P* = 0.13), and IFN-γ + TNF-α + IL-17A-(*P* = 0.00042 or *P* = 0.0075) secreting antigen-specific CD4^+^ or CD8^+^ T cells in spleen correlated with decrease bacterial burden in the liver (Fig. 7b), and the level of IFN-γ (*P* = 0.012), TNF-α (*P* = 0.00071), and IFN-γ + TNF-α (*P* = 0.0047) in serum also correlated with decreased bacterial burden in the liver (Fig. 7d). Consistent results were also seen in the intestine (Supplementary Fig. 1), suggesting the importance of IFN-γ, TNF-α, and IL-17A for protection against MAP.

## Discussion

The development of recombinant-protein subunit vaccines composed of clearly defined compositions produced by heterogeneous expression systems has received increasing attention in recent years^33,34^. A subunit vaccine cannot replicate in the body and thus is considered safer^35^. From a compatibility perspective, an ideal subunit vaccine would not interfere with diagnosis of disease by current tests. It has been found that several MAP proteins induce different levels of protection against infection in experimental animals^19,36,37^. Reports suggest that a mixture of recombinant antigens is better for vaccine development. However, the cost of preparing and using such a vaccine for cattle or sheep may be too high. To address this issue, fusion proteins are being constructed and tested for their ability to provide immune protection against MAP using a mouse model. A fusion protein, 74 F of MAP, with Monophosphoryl lipid A (MPL) and dimethyl octadecyl ammonium bromide (DDA) adjuvant has been found to protect mice and goats, respectively^21,24^. Inspired by the success of this fusion protein, we constructed four fusion proteins using the genes *map3527*, *ag85b*, and *hsp 70* of MAP (Fig. 1a). Veterinary vaccine costs are an essential topic, and using expensive immunostimulants, such as DDA and MPL, for large-scale production of animal vaccines would be prohibitive. In this study, we proposed using mineral oil adjuvant (Montanide ISA 61VG and Montanide ISA 206 VG) and water-based adjuvant (Montanide GEL 02 PR) as adjuvants in subunit vaccines since they can induce both specific humoral and cellular immune responses, and are widely used in vaccine research in bovine, sheep, and mice^38–41^. Although non-absorbable and strongly irritant, which are disadvantages, oil-based adjuvants can protect the antigen from rapid degradation by enzymes and the emulsion increases antigen persistence to induce sustained immune responses^38^.

IFN-γ is a major cytokine triggered when cattle are infected with MAP^42^, and studies have shown that increased expression of IFN-γ in lambs immunized with DNA vaccines can protect them from MAP infection^37^. In the present study, we immunized mice with the four fusion proteins (66NC, 66CN, 90NC, and 90CN) in combination with different adjuvants. Through the utilization of an IFN-γ ELISpot screen, we identified the most effective combination of immunogen (66NC) and Montanide ISA 61VG adjuvant. We observed that mice vaccinated with the 66NC fusion protein formulated with 61VG adjuvant had a significantly higher antigen-specific IFN-γ response than those immunized with adjuvant alone or 74 F fusion protein. Cell-mediated immune responses are thought to be the most successful in eliminating intracellular pathogens, including mycobacteria, and providing protection^43^. However, research has indicated that a vaccine comprising multiple components, which induces both T- and B-cell mediated immune responses, is more effective in protecting from *Mycobacterium tuberculosis* (MTB) H37Rv than a subunit vaccine that only stimulates T-cell immunity^44^. The current study revealed that mice immunized with the 66NC fusion protein had a more pronounced antigen-specific IL-4 response and a stronger antibody response than those immunized with adjuvant alone or 74 F. In addition, antibody during latent tuberculosis infection promoted phagolysosomal maturation and inflammasome activation, facilitating macrophage killing of intracellular MTB^45^. Furthermore, a strong initial IgG1 response following vaccinated sheep was crucial to prevent MAP infection^32^. Although a Th1 response generated after BCG vaccination does not always indicate protection^46^, IL-4, a Th2 cytokine, has been linked to defense against mycobacteria^47^. Overall, the 66NC fusion protein has been observed to elicit antigen-specific cell-mediated and humoral immune responses, which could be a significant factor in protection against MAP.

In the initial stages of MAP infection, CD4^+^ lymphocytes stimulate IFN-γ, activate macrophages in a classical manner (M1), release pro-inflammatory cytokines such as TNF-α, IL-1β, and IL-6, and enhance microbicidal activity^48–50^. During MAP infection, CD8^+^ cytotoxic T cells play a crucial role in the elimination of intracellular MAP^1^. We found that antigen-specific CD4^+^ or CD8^+^ T cell responses secreting IFN-γ and TNF-α were associated with protection in C57BL/6 mice after being immunized and challenged for two weeks. Furthermore, similar results were seen in the level of IFN-γ and TNF-α in the serum of immunized mice against MAP. We also observed that IL-17A-secreting antigen-specific CD4^+^ or CD8^+^ T cell responses after vaccination correlated with immunoprotection in C57BL/6 mice two weeks post-challenge. Evidence suggests that IL-17 secretion, which can be partly attributed to gamma-delta T (γδT) cells and Group 3 innate lymphoid (ILC3) cells, can enhance phagocytic capacity and antigen presentation^51,52^. Following vaccination with an attenuated MTB ΔLprG vaccine, increase of IL-17A levels in serum was associated with a decrease in bacterial burden in the lungs after the MTB challenge^53^. Analysis of the inactivated vaccine Silirum revealed that there was no statistically significant difference in the transcription levels of IL-17A between vaccinated and non-vaccinated goats by monocyte-derived macrophages^54^. Our study revealed that the serum of mice immunized with 66NC had a significantly higher level of IL-17A compared to the non-immunized and 74 F immunized groups. Despite the inability to detect IL-17A in serum two weeks post-challenge, antigen-specific CD4+ or CD8 + T cell responses that secrete IL-17A could still be observed. The main explanation for this is that IL-17 was observed to be reduced in PBMCs from both goats infected with MAP^28^ and cattle with a subclinical infection of MAP^55^. Additionally, the secretion of IL-17A cytokine may decrease in immunized mice against MAP infection, but concentration sensitivity of ELISA assay is limited.

In the absence of clinical symptoms, the gold standard for evaluating the protective effects of MAP vaccinations is the pathology and bacterial load of tissues^56,57^. Therefore, to evaluate the protective efficacy of 66NC, we chose the liver and intestine as the organs of assessment, as these were found to be reliable indicators of the infectious status of mice post-challenge with MAP in prior studies^26,58^. Two weeks after the challenge, a reduction in the number of CFU in both the liver and intestine was observed, implying the elimination of MAP. Furthermore, the 66NC fusion protein successfully reduced the pathological damage and quantity of acid-fast bacteria in the livers of vaccinated animals. Ruminants suffering from PTB experience diarrhea and a drastic reduction in body weight^59,60^. We measured the body weight of the 66NC vaccinated mice following the challenge. The body weight of the mice immunized with 66NC remained comparable to that of the blank mice, in contrast to the body weight of the mice immunized with 74 F and the adjuvant group, which decreased to different extents. In conclusion, immunization with 66NC was demonstrated to be significantly more effective in protecting mice against MAP infection than the 74 F vaccine, as evidenced by the substantially reduced MAP load in the organs, improved liver and intestine pathology, and maintenance of body weight.

In summary, vaccination with the 66NC fusion protein resulted in bacterial control, alleviated pathological damage in the liver and intestine, and reduced body weight loss following the MAP K-10 challenge in C57BL/6. A correlation was observed between protection and the secretion of IFN-γ, TNF-α, and IL-17A by antigen-specific CD4+ and CD8 + T lymphocytes in the spleens of mice after the challenge. Additionally, serum IFN-γ and TNF-α levels following immunization were linked to protective efficacy. Furthermore, mice immunized with 66NC demonstrate a greater protective effect against MAP K-10 than those immunized with 74 F. The distinct characteristics of the antigens in the fusion protein vaccine makes it unlikely to interfere with the bovine tuberculosis skin test in cattle or sheep. Additional research in species such as cattle or sheep is required to ascertain if the fusion protein 66NC subunit vaccine can protect against PTB without eliciting reactions to the bovine tuberculosis skin test.

## Methods

### Bacterial strains and animals

*Mycobacterium avium* subspecies *paratuberculosis* K-10 was grown in Middlebrook 7H9 medium (BD Biosciences, San Jose, CA, USA) or Middlebrook 7H10 medium (BD Biosciences) supplemented with 0.05% Tween 80 (Amresco, Solon, OH, USA), 0.2% glycerol (Sigma-Aldrich, Shanghai, China), 10% oleic acid-albumin-dextrose-catalase (OADC, BD Biosciences) and 2 mg/L mycobactin J (Allied Monitor, Fayette, MO, USA) at 37 °C. Six-week-old female C57BL/6 mice were purchased from Liaoning Changsheng Biotechnology Co. Ltd (Liaoning, China). All of the animal experiments were designed to minimize the number of animals used, and efforts were made to minimize both distress and pain. This study was approved by the Animal Ethics committee of Harbin Veterinary Research Institute, China (Ethical Committee Approval number HVRI-IACUC-200723-01).

### Generation of a tandemly linked ORF encoding 66NC, 66CN, 90NC, and 90CN

66NC was generated by the continuous connection in tandem with the open reading frames (ORFs) of the ~18.9 kDa N-terminal portion of MAP3527 (residues 40–232, 193 aa) to the ~30.8 kDa of Ag85B without the signal peptide (residues 41–330, 290 aa, with stop codon omitted), followed at the C terminus with the ~13.0 kDa C-terminal fragment (residues 231–361, 131 aa, with stop codon omitted) of MAP3527. In addition, 66NC comprises a single ORF organized in the MAP3527_N40–232_-Ag85B_41-330–_MAP3527_C231–361_ linear order; 66CN was generated by the tandem ORFs of the MAP3527_C231–361_ to the Ag85B followed with the MAP3527_N40–232_, from the MA3527_C231–361_- Ag85B_41–330_-MAP3527_N40–232_ ORF.

90NC, which includes the full-length ORF of Hsp70 (without stop codon) instead of Ag85b, is encoded by the MA3527_N40–232_-Hsp70-MAP3527_C231–361_ ORF. Likewise, 90CN includes the Hsp70 instead of Ag85b, but it is encoded by the MA3527_C231–361_-Hsp70-MAP3527_N40–232_ ORF.

### Construction of recombinant pET28a-MAP3527_N_-MAP3527_C_ and pET28a-MAP3527_C_-MAP3527_N_ plasmids

The map3527_N40–232_ and map3527_C231–361_ genes were amplified from the genome of MAP K-10 using PrimeSTAR Max DNA Polymerase (TaKaRa Bio, Beijing, China) and the MAP3527_N_-F/R and MAP3527_C_-F/R primers, respectively (Supplementary Table 1). The PCR reaction procedure is as follows to make it. Pre-degeneration at 98 °C for 5 min, degeneration at 98 °C for 10 s, annealing at 65 °C for 15 s, extension at 72 °C for 10 s, with 30 cycles, then final extension at 72 °C for 1 min. The PCR products were purified using PCR purification kit (TIANGEN, Beijing, China). They were then separately ligated into the pET28a vector (Novagen, Darmstadt, HE, Germany), which had been double-digested by *Nde* I and *Eco*R I, using the Trelief SoSoo Cloning Kit (TSINGKE, Beijing, China) at 50 °C for 15 min. The ligated products were then transformed into *Escherichia coli* (*E. coli*) DH5α and the colonies with the correct plasmids, which were named pET28a-MAP3527_N_ and pET28a-MAP3527_C_, were determined by PCR and DNA sequencing. Then map3527_N40–232_ and map3527_C231–361_ genes were again amplified using the MAP3527_NN_-F/R and MAP3527_CC_-F/R primers, respectively (Supplementary Table 1) and ligated into the pET28a-MAP3527_C_ and pET28a-MAP3527_N_, which had been double-digested by *Hin*d III and *Xho* I, using the Trelief SoSoo Cloning Kit. Finally, the ligated products were identified by PCR and verified by DNA sequencing and were named pET28a-MAP3527_N_-MAP3527_C_ and pET28a-MAP3527_C_- MAP3527_N_.

### Cloning of the ag85b and hsp70 into the pET28a-MAP3527_N_-MAP3527_C_ and pET28a-MAP3527_C_-MAP3527_N_ construct

The ag85b (without 1–40 aa signal peptide sequence) and hsp70 genes were amplified using the MAP K-10 genome as a template with Ag85B_NC_-F/R, Ag85B_CN_-F/R, Hsp70_NC_-F/R, and Hsp70_CN_-F/R primers, and subcloned into pET28a-MAP3527_N_-MAP3527_C_ and pET28a-MAP3527_C_-MAP3527_N_ constructs, which were double-digested by *Eco*R I and *Hin*d III, using Trelief SoSoo Cloning Kit. Following the ligation, the products were transformed into *E. coli* DH5α, and transformants containing the correct insert and orientation were verified by PCR and confirmed by DNA sequencing. The final constructs, encoding 66 kDa (66NC and 66CN) and 90 kDa (90NC and 90CN) fusion proteins, comprise a single ORF organized in the following linear orders: MAP3527_N_-Ag85B-MAP3527_C_, MA3527_C_-Ag85B-MAP3527_N_, MA3527_N_-Hsp70-MAP3527_C_, and MA3527_C_-Hsp70-MAP3527_N_.

### Recombinant protein expression and purification

The recombinant plasmid was expressed in *E. coli* BL21 strain (DE3) in LB medium containing 50 μg/mL kanamycin. An overnight grown culture of BL21 containing recombinant plasmid was added to 1 L of LB medium containing 50 μg/mL kanamycin and grown at 37 °C with shaking. Cultures were induced at an OD_600 nm_ of 0.6–0.8 with 1 mM isopropyl-*β*-D-thiogalactoside (IPTG) for 4 h at 37 °C, 350 × g. The induced bacterial cells were harvested and sonicated with buffer A (20 mM Tris-HCl-150 mM NaCl-10% glycerol-pH 8.0) and centrifuged at 33,264 × g for 10 min. Pellets were dissolved in buffer A containing 0.5% sodium lauroyl sarcosine and then dialyzed for 24 h against buffer A containing 0.1 mM oxidized glutathione, and 0.9 mM reduced glutathione. The protein that underwent dialysis was then centrifuged at a speed of 33,264 × g for 30 min, and the supernatant was loaded onto Ni-NAT resin (GE Healthcare Life Sciences, Uppsala, Sweden). Each target protein was eluted with elution buffer (20 mM Tris-HCl-150 mM NaCl-5% glycerol-500 mM imidazole-pH 8.0). Each purified protein was subjected to a ToxinEraser Endotoxin removal kit (Genscript, Nanjing, China) for removing endotoxin and confirmed by mass spectrometry analysis.

### Construction, expression, and purification of polyprotein 74 F

The construction procedure of the 74 F was referred to in the previous reports^21^. Brief descriptions are included below. The correct MAP3527_C183–361_ gene product was ligated into the pET17b vector, followed by the ligation of the MAP1519_N1–460_ product into the pET17b-MAP3527_C183–361_. Finally, the MAP3527_N33–180_ product was ligated into the pET17b-MAP3527_C183–361_-MAP1519_N1–460._ The recombinant plasmid (pET17b-MAP3527_C183–361_-MAP1519_N1–460_-MAP3527_N33–180_) was analyzed by sequencing and was transformed into the *E. coli* BL21 in LB medium containing 100 μg/mL of ampicillin. Expression and purification of 74 F were performed according to the methods described above. The confirmation of the purified 74 F protein was achieved through the use of mass spectrometry analysis.

### Screening adjuvant, immune pathway, and immunogenic protein

Thirty-six mice were immunized (in groups of three) with 50 μg of the 66NC fusion protein mixed with Montanide ISA 61 VG (water-in-oil, Seppic, Paris, France), Montanide ISA 206 VG (water-in-oil-in-water, Seppic), or Montanide GEL 02 PR (water-based adjuvant, Seppic) adjuvants at a protein or PBS solution: adjuvant volume ratio of 1:1.5, respectively. The same volume of PBS was separately mixed with the above three adjuvants as the negative control. The six groups mentioned above were vaccinated using two routes of immunization. Each group was administered twice, three weeks apart, in a total volume of 100 μL. Eighteen mice were injected intramuscular (IM) in the thigh, and another eighteen mice were injected subcutaneous (SC) on the back (Supplementary Table 2). Fifteen mice were immunized (in groups of three) with a total volume of 100 μL with Montanide ISA 61 VG adjuvant plus 50 μg of 66NC, 66CN, 90NC, or 90CN fusion protein or PBS by SC injection at a protein or PBS solution: adjuvant volume ratio of 1:1.5, respectively.

All animals in each group were euthanized by CO_2_ inhalation three weeks after the second immunization, and lymphocytes were isolated from the spleen using the Mouse Spleen Lymphocyte Separation Medium Kit (TBD Science, Tianjin, China) according to the manufacturer’s instructions for IFN-γ ELISpot assay. The details of the procedure are as follows. The mouse spleen was aseptically separated using scissors, cut into small pieces, and ground in the homogenate rinse fluid (TBD Science). The single-cell suspension was obtained through a 70-mesh screen, and then centrifuged at 350 × g for 10 min and the supernatants were discarded. All tissue cells were resuspended in tissue sample diluent (TBD Science) and then added onto the surface of lymphocyte separation medium (TBD Science). The spleen lymphocytes were obtained by centrifugation at 400 × g for 30 min and then washed with a wash solution (TBD Science) and purified by removing the adhering cells.

### Immunization and challenge of mice

Ninety-six mice were randomly separated into four groups and immunized subcutaneously twice at an interval of three weeks. Group I (six mice) was not subjected to any treatment, serving as blank control for body weight monitoring. Group II (30 mice) served as the unvaccinated group of mice that were administered Montanide ISA 61 VG adjuvant alone. Group III (30 mice) was composed of mice immunized twice with 50 μg/mouse of 66NC fusion protein mixed with Montanide ISA 61 VG adjuvant. Group IV (30 mice) immunized with 50 μg/mouse of 74 F and combined with MPL adjuvant (Sigma Adjuvant System, Shanghai, China) at a protein: adjuvant volume ratio of 1:1 and was used as the subunit vaccine control^21^. Splenocytes and serum samples from six mice in groups II, III, and IV were harvested three weeks after the secondary immunization for analysis by ELISpot, ELISA, and intracellular cytokine staining to evaluate immunogenicity as described hereafter.

The remaining mice in each group (*n* = 24) were challenged with 10^9^ colony-forming units (CFU)/mouse of MAP K-10 by intraperitoneal injection. Six mice per group were euthanized by CO_2_ inhalation at 2, 4, 8, and 12 weeks post-challenge, and livers and intestines were collected using sterile scissors, observed, and photographed for pathology examination and divided into two parts. One part was utilized for colony counting, while the other was perfused with 10% formaldehyde for histopathology analysis. In addition, splenocytes and serum samples of six mice per group were collected at two weeks post-challenge to analyze intracellular and secreted cytokines, respectively.

### ELISpot assay

Antigen-specific immune responses to 66NC or 74 F were assessed by IFN-γ and IL-4 ELISpot assays according to Liu J et al. with minor modifications^61^. Details of methods are as follows. Isolated splenic lymphocytes (5 × 10^5^ cells per well) were added into precoated 96-well plates with anti-IFN-γ or IL-4 monoclonal antibody and stimulated at 37 °C for 48 h with 10 μg/mL 66NC or 74 F fusion protein. Same volume of PBS was added as the negative control. The numbers of IFN-γ- and IL-4-secreting splenocytes were measured using mouse IFN-γ or IL-4 ELISpot kit (Dakewe, Beijing, China), respectively, according to the manufacturer’s instructions. The plates were air-dried, and spots were counted using an iSpot Reader Spectrum (AID, Strassberg, Germany).

### Enzyme-linked immunosorbent assay (ELISA) for antibody level

Antigen-specific IgG, IgM, IgG1, and IgG2a levels were determined by a traditional sandwich ELISA. Costar 96-well ELISA plates (Corning, NY, USA) were coated with 66NC or 74 F (500 ng/well) in a sodium hydrogen carbonate buffer (CBS, pH 8.5) at 4 °C overnight. Then, to prevent nonspecific protein binding, 5% skim milk was added to PBST (0.05% Tween-20 in PBS, pH 7.4) for 2 h at 37 °C. Following three washes with PBST, serial dilutions of serum samples (blood was collected from the tail vein of mice and centrifuged at 1000 × g for 5 min) were added to the antigen-coated ELISA plates, which were incubated at 37 °C for 1 h. After washing, horseradish peroxidase (HRP) conjugated rabbit or goat anti-mouse IgG (ab6728)/IgM (ab97230)/IgG1 (ab97240)/IgG2a (ab97245) (1:10,000 dilution in PBST, Abcam, Cambridge, MA, USA) were used as the secondary antibodies and incubated at 37 °C for 1 h. Finally, the plates were washed, and a TMB (Sigma-Aldrich, Shanghai, China) substrate was added and incubated for 15 min for color development. The readout was carried out at OD450_nm_ using an ELx808 microplate reader (BioTek Instruments, Winooski, VT, USA) after adding 2 M H_2_SO_4_ as a stop solution. The serum antibody titer is the largest dilution at which the OD450_positive_/OD450_negative_ > 2.1.

### Intracellular cytokine staining and ELISA for cytokine secretion

Intracellular cytokine staining (ICS) assays were used to evaluate antigen-specific CD4^+^ and CD8^+^ T-lymphocyte responses and performed as previously described with slight modification^61^. Briefly, 1.5 × 10^6^ splenocytes were seeded to a 6-well plate and cultured at 37 °C with a final concentration of 10 μg/mL 66NC or 74 F fusion protein. The same volume of PBS was added as the control. After 12 h incubation, Golgi-Plug and Golgi-Stop (BD Biosciences) were added. Cells were incubated for 4 h. Cells were stained with PE-conjugated Anti-Mouse CD4 (12-0041-82)/CD8α (MCD0804) antibody (1:50, ThermoFisher, Waltham, MA, USA) at 4 °C for 30 min, protected from light. After washing twice with cold FACS buffer, the cells were fixed and permeabilized with Cytofix/Cytoperm (BD Biosciences), and then washed twice with Perm Wash buffer. Cells were stained with PE/Cy5-conjugated Anti-IFN-γ (1:50, XMG1.2, ab272255, Abcam), Alexa Fluor 647-conjugated Rat anti-Mouse IL-17A (1:50, TC11-18H10, 560184, BD Biosciences), eFluor 450-conjugated anti-Mouse TNF-α (1:50, MP6-XT22, 48-7321-82, ThermoFisher) at 4 °C for 30 min, shaded from the light. Antibody PE/Cy5 Rat IgG1 (1:50, RG1, NBP1-43076, Novus Biologicals, Littleton, Co, USA), Alexa Fluor 647 Rat IgG1 (1:50, KLH/G1-2-2, 0116-31, SouthernBiotech, Birmingham, AL, USA), and eFluor 450 Rat IgG1 (1:50, eBRG1, 48-4301-82, ThermoFisher) were used according to the manufacturer’s instructions for isotype control. Cells were washed twice with Perm Wash buffer, resuspended in PBS, detected using an Apogee A50-Micro Nanoscale Flow Cytometry (Apogee Flow Systems, London, UK), and analyzed with Apogee Histogram software.

Serum samples were collected at three weeks post-secondary immunization and two weeks after challenge and compared to non-vaccinated mice. According to the manufacturer’s instructions, the serum cytokines were determined by IFN-γ/TNF-α/IL-17A Mouse ELISA Kit (ThermoFisher). In addition, multiple regression analyses of serum and intracellular cytokines levels with CFU in the liver and intestine were performed using the R ggplot2 and ggpubr packages.

### Tissue CFU enumeration, macropathology, and histopathology

Livers and intestines from MAP-challenged mice were harvested under aseptic conditions 2, 4, 8, and 12 weeks after MAP challenge and washed with PBS. The livers and intestines were photographed with a digital camera (Canon, Tokyo, Japan) for macropathology analysis. One-half of the liver and intestine were used to enumerate MAP CFU. They were homogenized with a sterile PBST solution, followed by serial dilutions onto 7H10 plates containing 2 mg/L mycobactin J and incubated at 37 °C for 3–4 weeks. And the other half of the liver and intestine were perfusion-fixed in 10% neutral formalin, dehydrated, embedded in paraffin blocks, and sectioned for hematoxylin and eosin (H&E) Ziehl-Neelsen acid-fast stains. Images were accomplished by a Nikon Eclipse E100 microscopy (Nikon, Tokyo, Japan). Two independent pathologists quantified lung and intestine injury scoring in a double-blind manner. For macropathology scoring, the percentage of granuloma area on the liver surface was scored from 0–4 using the following scale: 0 = normal (no granuloma), 1 = slightly increased (<1%), 2 = moderately increased (≥1% and <3%), 3 = greatly increased (≥3%), 4 = greatly increased (>3%) with prominent granulomas. The number of granulomas in the intestine surface was scored from 0 to 3 using the following scale: 0 = no granuloma, 1 = one granuloma, 2 = two granuloma, and 3 = three granulomas. For histopathology scoring, the severity of liver lesions was scored from 0 to 4 using the following scale: 0 = normal, 1 = multiple micro-granulomas, 2 = multiple micro-granulomas with focal necrosis, 3 = macro-granulomas, 4 = macro-granulomas with massive necrosis. The severity of intestine lesions was scored from 0 to 3 using the following scale: 0 = normal, 1 = multiple microgranulomas, 2 = macro-granulomas, 3 = macro-granulomas, and inflammatory cell infiltration.

### Statistical analysis

One-way and two-way ANOVA were used to analyze statistical significance, followed by Tukey’s multiple comparisons tests. All statistical analyses were performed using GraphPad Prism 9.0.0. A *P* value < 0.05 was statistically significant (*P < 0.05; **P < 0.01; ***P < 0.001; ****P < 0.0001).

### Reporting summary

Further information on research design is available in the Nature Research Reporting Summary linked to this article.

## Supplementary information

Supplementary Information

REPORTING SUMMARY

## Data Availability

All data associated with this study are present in the paper or the Supplementary Information. The resources, data, and reagents of the current study are available from the corresponding author upon reasonable request.
