# Supplementary material for: A candidate subunit vaccine induces protective immunity against Mycobacterium avium subspecies paratuberculosis in mice
Source: NPJ Vaccines. 2023 May 20;8:72. doi: 10.1038/s41541-023-00675-1 (PMC10199288; doi:10.1038/s41541-023-00675-1)
Supplement: Supplementary file 1 — Supplementary Information [file 41541_2023_675_MOESM1_ESM.pdf]

Supplementary Materials

Supplementary Table 1. Primers used to amplify *map*3527, *ag85b* and *hsp70* genes

Primers	Sequence（5ʹ -3ʹ）	Restriction site
MAP3527$_{C}$-F:	CGCGCGGCAGCCATATGACCGCCGCCACCGACAGCTACA	*Nde* I
MAP3527$_{C}$-R:	TCGACGGAGCTCGAATTCGGCCGGCGGCCCCTCCGCCAG	*Eco*R I
MAP3527$_{NN}$-F:	GCTCCGTCGACAAGCTTGCGCACCGTCGGGCCTGGCGCT	*Hin*d III
MAP3527$_{NN}$-R:	GTGGTGGTGGTGCTCGAGTGGCGGTGTCCACGCCGATCACCT	*Xho* I
MAP3527$_{N}$-F:	GCGCGGCAGCCATATGGCACCGTCGGGCCTGGCGCTGGA	*Nde* I
MAP3527$_{N}$-R:	CGACGGAGCTCGAATTCGGCGGTGTCCACGCCGATCACC	*Eco*R I
MAP3527$_{CC}$-F:	AGCTCCGTCGACAAGCTTGCACCGCCGCCACCGACAGCT	*Hin*d III
MAP3527$_{CC}$-R:	TGGTGGTGGTGCTCGAGTGGCCGGCGGCCCCTCCGCCA	*Xho* I
Ag85B$_{CN}$-F:	GGCCGCCGGCCGAATTCTTTTCGCGTCCGGGCCTGCCC	*Eco*R I
Ag85B$_{CN}$-R:	GACGGTGCGCAAGCTTGTCCGCCGCCGCCCGGGGACG	*Hin*d III
Ag85B$_{NC}$-F:	GCGTGGACACCGCCGAATTCTTTTCGCGTCCGGGCCTGCCCG	*Eco*R I
Ag85B$_{NC}$-R:	CGGCGGTGCAAGCTTGTCCGCCGCCGCCCGGGGACG	*Hin*d III
Hsp70$_{CN}$-F:	AGGGGCCGCCGGCCGAATTCATGAGAGTCGGAATCGACTTCGGC	*Eco*R I
Hsp70$_{CN}$-R：	CCCGACGGTGCGCAAGCTTGGCTCGCCGATGCGCACCGC	*Hin*d III

	TC	
Hsp70$_{NC}$-F:	CGGCGTGGACACCGCCGAATTCATGAGAGTCGGAATCGA CTTCGGC	*Eco*R I
Hsp70$_{NC}$-R:	GGTGGCGGCGGTGCAAGCTTGGCTCGCCGATGCGCACCG CTC	*Hin*d III

Supplementary Table 2. Experimental grouping of mice for screening adjuvants and immune pathways (36 mice, n=3 per group)

	Adjuvant \ Immune pathway	IM (18 mice)	SC (18 mice)
PBS+	Montanide ISA 61 VG	Group I	Group VII
	Montanide ISA 206 VG	Group II	Group VIII
	Montanide GEL 02 PR	Group III	Group IX
66NC+	Montanide ISA 61 VG	Group IV	Group X
	Montanide ISA 206 VG	Group V	Group XI
	Montanide GEL 02 PR	Group VI	Group XII

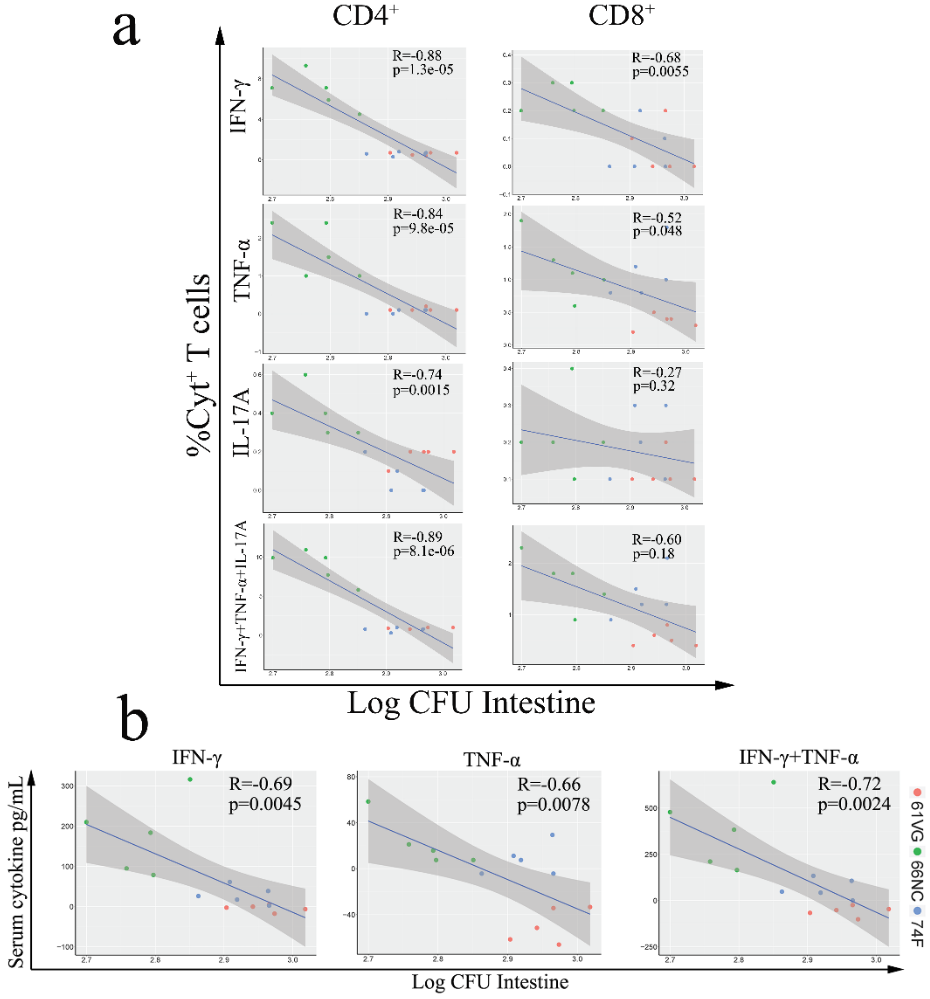

Supplementary Figure 1. Cellular immune responses post MAP challenge of 66NC vaccinated C57BL/6 mice. (**a**) Pearson's correlation (R) of Cyt^{+} CD4^{+} and CD8^{+} T lymphocytes with CFU in the intestine following challenge. (**b**) Pearson's correlations of cytokine levels with CFU in the intestine following challenge for IFN-γ and TNF-α.

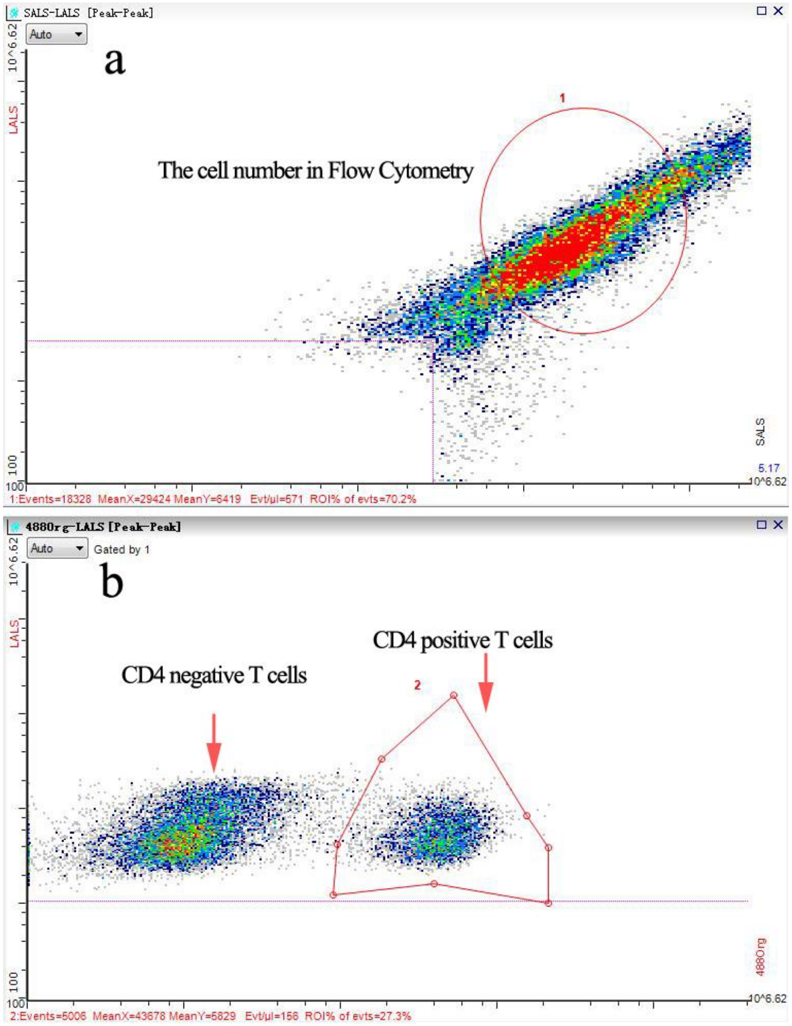

Supplementary Figure 2. An example of the gating strategy in flow cytometry

Supplementary Sequence 1. The nucleotide and amino acid sequence of four fusion protein (66CN, 66NC, 90CN, and 90NC).

1-1. The nucleotide sequences of 66CN (1902bp)

ATGACCGCCGCCACCGACAGCTACAAGATGTCCGGCGGGCAGGGCTTCGCCATCCCGA
TCGGCCGCGCCATGGCCGTCGCCAACCAGATCCGCTCCGGCGCCGGCTCCAACACCGT
GCACATCGGGCCCACCGCATTCCTCGGGCTGGGCGTGACGGACAACAACGGCAACGG
CGCGCGGGTGCAGCGGGTGGTCAACACCGGCCCGGCCGCGGCCGCGGGCATCGCGCC
CGGCGACGTCATCACCGGCGTCGACACCGTCCCGATCAACGGGGCGACGTCGATGAC
CGAGGTGCTCGTCCCGCACCACCCCGGTGACACCATCGCGGTGCACTTCCGGTCCGTC
GACGGCGGCGAGCGCACCGCGAACATCACCCTGGCGGAGGGGCCGCCGGCCGAATTC
TTTTCGCGTCCGGGCCTGCCCGTCGAGTACCTGCAGGTGCCCTCCGCGGGAATGGGCC
GCGACATCAAGGTCCAGTTCCAGAGCGGCGGCAACGGCTCCCCCGCGGTGTATCTGCT
GGACGGCCTGCGGGCTCAGGACGACTACAACGGCTGGGACATCAACACCCCGGCCTT

CGAGTGGTACTACCAGTCCGGCCTGTCGGTGATCATGCCCGTCGGCGGACAGTCCAGC
TTCTACGCGGACTGGTACCAGCCCGCGTGCGGCAAGGCCGGTTGCTCCACTTATAAGT
GGGAGACCTTCCTGACCAGCGAGCTGCCGTCGTACCTGGCCTCCAACAAGGGTGTGA
AGCGCACCGGCAGCGCCGCAGTCGGCATTTCGATGTCCGGATCCTCGGCGATGATCCT
GGCCGTCAACCATCCCGACCAATTCATCTATGCCGGATCGCTCTCGGCGCTGCTCGACC
CGTCCCAGGGCATGGGGCCGTCGCTGATCGGTCTGGCGATGGGTGACGCCGGCGGCTA
CAAGGCCGACGCCATGTGGGGCCCGTCCAGCGACCCGGCCTGGCAGCGCAACGACCC
GAGCCTGCACATCCCCGAGCTGGTCGGCCACAACACCCGGCTGTGGGTGTACTGCGGT
AACGGGACACCGTCGGAGCTGGGTGGCGCCAACATGCCCGCCGAGTTCCTGGAGAAC
TTCGTGCGCAGCAGCAACCTGAAGTTCCAGGACGCCTACAACGCCGCCGGCGGCCAC
AACGCCGTGTTCAACTTCAACGCCAACGGAACGCACAGCTGGGAGTACTGGGGAGCC
CAGCTCAACGCCATGAAGCCCGACCTGCAGGGCACCCTGGGCGCGTCCCCGGGCGGC
GGCGGACAAGCTTGCGCACCGTCGGGCCTGGCGCTGGACCGGTTCGCCGATCGCCCC
CTGGCGCCGATCGACCCGTCGGCCATGGTCGGTCAGGTGGGGCCGCAAGTGGTCAAC
ATCGACACCAAGTTCGGCTACAACAACGCGGTGGGCGCCGGTACCGGCATCGTGATCG
ACCCGAACGGCGTGGTGCTCACCAACAACCACGTCATCTCGGGCGCCACCGAAATCA
GCGCGTTCGACGTCGGCAACGGGCAGACCTACGCCGTCGACGTGGTCGGCTATGACC
GCACCCAGGACATCGCCGTGCTGCAGCTGCGCGGCGCGGCCGGCCTGCCCACCGCCA
CCATCGGCGGCGAGGCCACGGTGGGCGAGCCCATCGTCGCGCTTGGCAACGTCGGCG
GCCAGGGCGGCACCCCCAACGCGGTGGCCGGCAAGGTCGTCGCGCTCAACCAGAGCG
TCTCGGCGACCGACACGCTGACCGGCGCGCAGGAGAACCTCGGCGGCCTGATCCAGG
CCGACGCGCCGATCAAGCCGGGCGACTCCGGTGGCCCGATGGTGAACAGCGCCGGGC
AGGTGATCGGCGTGGACACCGCCACTCGAGCACCACCACCACCACCACTGAGATCCG
GCTGCTAA

1-2. The amino acid sequences of 66CN (653aa)

MGSSHHHHHHSSGLVPRGSHMTAATDSYKMSGGQGFAIPIGRAMAVANQIRSGAGSNTV
HIGPTAFLGLGVTDNNGNGARVQRVVNTGPAAAAGIAPGDVITGVDTVPINGATSMTEVL
VPHHPGDTIAVHFRSVDGGERTANITLAEGPPAEFFSRPGLPVEYLQVPSAGMGRDIKVQF
QSGGNGSPAVYLLDGLRAQDDYNGWDINTPAFEWYYQSGLSVIMPVGGQSSFYADWYQP
ACGKAGCSTYKWETFLTSELPSYLASNKGVKRTGSAAVGISMSGSSAMILAVNHPDQFIYA
GSLSALLDPSQGMGPSLIGLAMGDAGGYKADAMWGPSSDPAWQRNDPSLHIPELVGHNT
RLWVYCGNGTPSELGGANMPAEFLENFVRSSNLKFQDAYNAAGGHNAVFNFNANGTHS
WEYWGAQLNAMKPDLQGTLGASPGGGGQACAPSGLALDRFADRPLAPIDPSAMVGQVG
PQVVNIDTKFGYNNAVGAGTGIVIDPNGVVLTNNHVISGATEISAFDVGNGQTYAVDVVG
YDRTQDIAVLQLRGAAGLPTATIGGEATVGEPIVALGNVGGQGGTPNAVAGKVVALNQSV
SATDTLTGAQENLGGLIQADAPIKPGDSGGPMVNSAGQVIGVDTATRAPPPPPLRSGC

2-1. The nucleotide sequences of 66NC (1902bp)

ATGGCACCGTCGGGCCTGGCGCTGGACCGGTTCGCCGATCGCCCCCTGGCGCCGATCG
ACCCGTCGGCCATGGTCGGTCAGGTGGGGCCGCAAGTGGTCAACATCGACACCAAGT
TCGGCTACAACAACGCGGTGGGCGCCGGTACCGGCATCGTGATCGACCCGAACGGCG
TGGTGCTCACCAACAACCACGTCATCTCGGGCGCCACCGAAATCAGCGCGTTCGACGT
CGGCAACGGGCAGACCTACGCCGTCGACGTGGTCGGCTATGACCGCACCCAGGACAT
CGCCGTGCTGCAGCTGCGCGGCGCGGCCGGCCTGCCCACCGCCACCATCGGCGGCGA

GGCCACGGTGGGCGAGCCCATCGTCGCGCTTGGCAACGTCGGCGGCCAGGGCGGCAC
CCCCAACGCGGTGGCCGGCAAGGTCGTCGCGCTCAACCAGAGCGTCTCGGCGACCGA
CACGCTGACCGGCGCGCAGGAGAACCTCGGCGGCCTGATCCAGGCCGACGCGCCGAT
CAAGCCGGGCGACTCCGGTGGCCCGATGGTGAACAGCGCCGGGCAGGTGATCGGCGT
GGACACCGCCGAATTCTTTTCGCGTCCGGGCCTGCCCGTCGAGTACCTGCAGGTGCCC
TCCGCGGGAATGGGCCGCGACATCAAGGTCCAGTTCCAGAGCGGCGGCAACGGCTCC
CCCGCGGTGTATCTGCTGGACGGCCTGCGGGCTCAGGACGACTACAACGGCTGGGAC
ATCAACACCCCGGCCTTCGAGTGGTACTACCAGTCCGGCCTGTCGGTGATCATGCCCGT
CGGCGGACAGTCCAGCTTCTACGCGGACTGGTACCAGCCCGCGTGCGGCAAGGCCGG
TTGCTCCACTTATAAGTGGGAGACCTTCCTGACCAGCGAGCTGCCGTCGTACCTGGCCT
CCAACAAGGGTGTGAAGCGCACCGGCAGCGCCGCAGTCGGCATTTCGATGTCCGGAT
CCTCGGCGATGATCCTGGCCGTCAACCATCCCGACCAATTCATCTATGCCGGATCGCTC
TCGGCGCTGCTCGACCCGTCCCAGGGCATGGGGCCGTCGCTGATCGGTCTGGCGATGG
GTGACGCCGGCGGCTACAAGGCCGACGCCATGTGGGGCCCGTCCAGCGACCCGGCCT
GGCAGCGCAACGACCCGAGCCTGCACATCCCCGAGCTGGTCGGCCACAACACCCGGC
TGTGGGTGTACTGCGGTAACGGGACACCGTCGGAGCTGGGTGGCGCCAACATGCCCG
CCGAGTTCCTGGAGAACTTCGTGCGCAGCAGCAACCTGAAGTTCCAGGACGCCTACA
ACGCCGCCGGCGGCCACAACGCCGTGTTCAACTTCAACGCCAACGGAACGCACAGCT
GGGAGTACTGGGGAGCCCAGCTCAACGCCATGAAGCCCGACCTGCAGGGCACCCTGG
GCGCGTCCCCGGGCGGCGGCGGACAAGCTTGCACCGCCGCCACCGACAGCTACAAGA
TGTCCGGCGGGCAGGGCTTCGCCATCCCGATCGGCCGCGCCATGGCCGTCGCCAACCA
GATCCGCTCCGGCGCCGGCTCCAACACCGTGCACATCGGGCCCACCGCATTCCTCGGG
CTGGGCGTGACGGACAACAACGGCAACGGCGCGCGGGTGCAGCGGGTGGTCAACAC
CGGCCCGGCCGCGGCCGCGGGCATCGCGCCCGGCGACGTCATCACCGGCGTCGACAC
CGTCCCGATCAACGGGGCGACGTCGATGACCGAGGTGCTCGTCCCGCACCACCCCGGT
GACACCATCGCGGTGCACTTCCGGTCCGTCGACGGCGGCGAGCGCACCGCGAACATC
ACCCTGGCGGAGGGGCCGCCGGCCACTCGAGCACCACCACCACCACCACTGAGATCC
GGCTGCTAA

2-2. The amino acid sequences of 66NC (653aa)

MGSSHHHHHHSSGLVPRGSHMAPSGLALDRFADRPLAPIDPSAMVGQVGPQVVNIDTKF
GYNNAVGAGTGIVIDPNGVVLTNNHVISGATEISAFDVGNGQTYAVDVVGYDRTQDIAVL
QLRGAAGLPTATIGGEATVGEPIVALGNVGGQGGTPNAVAGKVVALNQSVSATDTLTGAQ
ENLGGLIQADAPIKPGDSGGPMVNSAGQVIGVDTAEFFSRPGLPVEYLQVPSAGMGRDIK
VQFQSGGNGSPAVYLLDGLRAQDDYNGWDINTPAFEWYYQSGLSVIMPVGGQSSFYADW
YQPACGKAGCSTYKWETFLTSELPSYLASNKGVKRTGSAAVGISMSGSSAMILAVNHPDQ
FIYAGSLSALLDPSQGMGPSLIGLAMGDAGGYKADAMWGPSSDPAWQRNDPSLHIPELVG
HNTRLWVYCGNGTPSELGGANMPAEFLENFVRSSNLKFQDAYNAAGGHNAVFNFNANG
THSWEYWGAQLNAMKPDLQGTLGASPGGGGQACTAATDSYKMSGGQGFAIPIGRAMAV
ANQIRSGAGSNTVHIGPTAFLGLGVTDNNGNGARVQRVVNTGPAAAAGIAPGDVITGVDT
VPINGATSMTEVLVPHHPGDTIAVHFRSVDGGERTANITLAEGPPATRAPPPPPLRSGC

3-1. The nucleotide sequences of 90CN (2547bp)

ATGACCGCCGCCACCGACAGCTACAAGATGTCCGGCGGGCAGGGCTTCGCCATCCCGA
TCGGCCGCGCCATGGCCGTCGCCAACCAGATCCGCTCCGGCGCCGGCTCCAACACCGT

GCACATCGGGCCCACCGCATTCCTCGGGCTGGGCGTGACGGACAACAACGGCAACGG
CGCGCGGGTGCAGCGGGTGGTCAACACCGGCCCGGCCGCGGCCGCGGGCATCGCGCC
CGGCGACGTCATCACCGGCGTCGACACCGTCCCGATCAACGGGGCGACGTCGATGAC
CGAGGTGCTCGTCCCGCACCACCCCGGTGACACCATCGCGGTGCACTTCCGGTCCGTC
GACGGCGGCGAGCGCACCGCGAACATCACCCTGGCGGAGGGGCCGCCGGCCGAATTC
ATGAGAGTCGGAATCGACTTCGGCACAACCCACACCGTCGTCGCCGCCGTCGACCGC
GGCAACTATCCGGTCGTCTTCTTCGACGGCATGGACGCGTGGCCGTCGATCATCGCCGC
CAACGCCGCCGGGGAGCTGCGCTACGGCGCGGACGCGGCCGCCGTGCGCCACGACCC
GTCGTGGTCGGTGCTGCGCTCGTTCAAACGTCTGCTCAACGACGCCGGGCCGCAGAC
CCGGGTCAAGCTGGCCGGCCGTGACTACCGGCTGGCCGAACTGCTGACCGGTTTTCTG
GCGCGGCTGAAAACCGATCTGCAGCAGCACTCCAACGCCACCGTGGCCCCCGGGGAG
CCCATCGAGGCCGCGATCAGCGTGCCGGCCAACGCCTCCAGCGCCCAGCGGCTGCTG
ACCCTGGACGCCTACGTGGCCGCCGGTTTCCACGTCGTCGCCCTGCTCAACGAGCCGT
CCGCGGCGAGCCTGGAATACGCGCATCGGTACCGCTCCACCATCACCGCCAAACGGGA
GTACGTGCTCGTCTACGACCTCGGCGGCGGAACCTTCGACGCGTCGCTGCTGAAGATG
ACCGGGCACTCCAACGAGGTGGTGGTCAGCGAGGGCATCCAGCGGCTCGGCGGCGAC
GACTTCGACGAGGCGATCGTCGAGCTGGTGCGGGCCGGTGCGGACCTGCCCGGCCTC
GACGCCGCCGGCCGCGCGTTGCTGGCCGAGGAATGCGCCGCCCGCAAGGAGGCGGTC
GGGCCGCAGACCCGCCGCTTCCTGGTTGACCTGTCGCCGTTCGACCGGCCCCCGTTCT
CCTGTCCGGTCGACGACGTGTACGCGGCGTGCGCCCCGCTGGTCGATCCGACGATGCG
GCTGCTGGAGCGGGTGCTGCGCGACCCGAGCCGCGGCCGCGCGGACGTGGACTGGTC
GGAGGTGGCCGGCATCTATGTGGTCGGCGGGGCCGGCGGCTTCCCGCTGGTGTCCCGG
ATGCTGCGCGCCCGGTTCGGGGACAAGCGGGTCAAGCGCTCACCGCACCCGTTCGCC
GCGACCGCCATCGGGTTGGCGGTGTTCTTGGACAAGGAATCGGGTTTTGCGCTGTCCG
AACGCTTTTCGCGGAATTTCGGGGTGTTCCGGGAGGCGGAGGCGGGCGCCGGGGTGG
TGTTCGACCCGATCGTGTGCAAGGACGTGTCGCTGCCGGCCGACGGGCAGACCCCGC
TGGTGGTCAAGCGGCGATACCGCGCCGCGCACAACATCGGGCACTTCCGGTTCGTGGA
GTGCAGCCGGCTGGTCGACGGGCGACCCGACGGCGACATCACCCCGTATGACCCGGT
GCTGTTTCCGTTCGACCCGGCGCTGCGCGACCGCGACGACCTGGGCCGCCAGCCGGT
CGGCCGGTGGCGGGACGGACCCGACGTCGAGGAGCGCTACGTGATCGCGCCCAGCGG
TGCGGTGGAGGTGACGTTGACGACGCAGCCGGCCGGCTTCGAACGCACCTTCCGGCT
GGAGCGGTGCGCATCGGCGAGCCAAGCTTGCGCACCGTCGGGCCTGGCGCTGGACCG
GTTCGCCGATCGCCCCCTGGCGCCGATCGACCCGTCGGCCATGGTCGGTCAGGTGGGG
CCGCAAGTGGTCAACATCGACACCAAGTTCGGCTACAACAACGCGGTGGGCGCCGGT
ACCGGCATCGTGATCGACCCGAACGGCGTGGTGCTCACCAACAACCACGTCATCTCGG
GCGCCACCGAAATCAGCGCGTTCGACGTCGGCAACGGGCAGACCTACGCCGTCGACG
TGGTCGGCTATGACCGCACCCAGGACATCGCCGTGCTGCAGCTGCGCGGCGCGGCCGG
CCTGCCCACCGCCACCATCGGCGGCGAGGCCACGGTGGGCGAGCCCATCGTCGCGCTT
GGCAACGTCGGCGGCCAGGGCGGCACCCCCAACGCGGTGGCCGGCAAGGTCGTCGC
GCTCAACCAGAGCGTCTCGGCGACCGACACGCTGACCGGCGCGCAGGAGAACCTCGG
CGGCCTGATCCAGGCCGACGCGCCGATCAAGCCGGGCGACTCCGGTGGCCCGATGGT
GAACAGCGCCGGGCAGGTGATCGGCGTGGACACCGCCACTCGAGCACCACCACCACC
ACCACTGAGATCCGGCTGCTAA

3-2. The amino acid sequences of 90CN (868aa)

MGSSHHHHHHSSGLVPRGSHMTAATDSYKMSGGQGFAIPIGRAMAVANQIRSGAGSNTV
HIGPTAFLGLGVTDNNGNGARVQRVVNTGPAAAAGIAPGDVITGVDTVPINGATSMTEVL
VPHHPGDTIAVHFRSVDGGERTANITLAEGPPAEFMRVGIDFGTTHTVVAAVDRGNYPVVF
FDGMDAWPSIIAANAAGELRYGADAAAVRHDPSWSVLRSFKRLLNDAGPQTRVKLAGRD
YRLAELLTGFLARLKTDLQQHSNATVAPGEPIEAAISVPANASSAQRLLTLDAYVAAGFHV
VALLNEPSAASLEYAHRYRSTITAKREYVLVYDLGGGTFDASLLKMTGHSNEVVVSEGIQ
RLGGDDFDEAIVELVRAGADLPGLDAAGRALLAEECAARKEAVGPQTRRFLVDLSPFDRP
PFSCPVDDVYAACAPLVDPTMRLLERVLRDPSRGRADVDWSEVAGIYVVGGAGGFPLVSR
MLRARFGDKRVKRSPHPFAATAIGLAVFLDKESGFALSERFSRNFGVFREAEAGAGVVFDP
IVCKDVSLPADGQTPLVVKRRYRAAHNIGHFRFVECSRLVDGRPDGDITPYDPVLFPFDPA
LRDRDDLGRQPVGRWRDGPDVEERYVIAPSGAVEVTLTTQPAGFERTFRLERCASASQAC
APSGLALDRFADRPLAPIDPSAMVGQVGPQVVNIDTKFGYNNAVGAGTGIVIDPNGVVLT
NNHVISGATEISAFDVGNGQTYAVDVVGYDRTQDIAVLQLRGAAGLPTATIGGEATVGEPI
VALGNVGGQGGTPNAVAGKVVALNQSVSATDTLTGAQENLGGLIQADAPIKPGDSGGPM
VNSAGQVIGVDTATRAPPPPPLRSGC

4-1. The nucleotide sequences of 90NC (2547bp)

ATGGCACCGTCGGGCCTGGCGCTGGACCGGTTCGCCGATCGCCCCCTGGCGCCGATCG
ACCCGTCGGCCATGGTCGGTCAGGTGGGGCCGCAAGTGGTCAACATCGACACCAAGT
TCGGCTACAACAACGCGGTGGGCGCCGGTACCGGCATCGTGATCGACCCGAACGGCG
TGGTGCTCACCAACAACCACGTCATCTCGGGCGCCACCGAAATCAGCGCGTTCGACGT
CGGCAACGGGCAGACCTACGCCGTCGACGTGGTCGGCTATGACCGCACCCAGGACAT
CGCCGTGCTGCAGCTGCGCGGCGCGGCCGGCCTGCCCACCGCCACCATCGGCGGCGA
GGCCACGGTGGGCGAGCCCATCGTCGCGCTTGGCAACGTCGGCGGCCAGGGCGGCAC
CCCCAACGCGGTGGCCGGCAAGGTCGTCGCGCTCAACCAGAGCGTCTCGGCGACCGA
CACGCTGACCGGCGCGCAGGAGAACCTCGGCGGCCTGATCCAGGCCGACGCGCCGAT
CAAGCCGGGCGACTCCGGTGGCCCGATGGTGAACAGCGCCGGGCAGGTGATCGGCGT
GGACACCGCCGAATTCATGAGAGTCGGAATCGACTTCGGCACAACCCACACCGTCGTC
GCCGCCGTCGACCGCGGCAACTATCCGGTCGTCTTCTTCGACGGCATGGACGCGTGGC
CGTCGATCATCGCCGCCAACGCCGCCGGGGAGCTGCGCTACGGCGCGGACGCGGCCG
CCGTGCGCCACGACCCGTCGTGGTCGGTGCTGCGCTCGTTCAAACGTCTGCTCAACGA
CGCCGGGCCGCAGACCCGGGTCAAGCTGGCCGGCCGTGACTACCGGCTGGCCGAACT
GCTGACCGGTTTTCTGGCGCGGCTGAAAACCGATCTGCAGCAGCACTCCAACGCCACC
GTGGCCCCCGGGGAGCCCATCGAGGCCGCGATCAGCGTGCCGGCCAACGCCTCCAGC
GCCCAGCGGCTGCTGACCCTGGACGCCTACGTGGCCGCCGGTTTCCACGTCGTCGCCC
TGCTCAACGAGCCGTCCGCGGCGAGCCTGGAATACGCGCATCGGTACCGCTCCACCAT
CACCGCCAAACGGGAGTACGTGCTCGTCTACGACCTCGGCGGCGGAACCTTCGACGC
GTCGCTGCTGAAGATGACCGGGCACTCCAACGAGGTGGTGGTCAGCGAGGGCATCCA
GCGGCTCGGCGGCGACGACTTCGACGAGGCGATCGTCGAGCTGGTGCGGGCCGGTGC
GGACCTGCCCGGCCTCGACGCCGCCGGCCGCGCGTTGCTGGCCGAGGAATGCGCCGC
CCGCAAGGAGGCGGTCGGGCCGCAGACCCGCCGCTTCCTGGTTGACCTGTCGCCGTT
CGACCGGCCCCCGTTCTCCTGTCCGGTCGACGACGTGTACGCGGCGTGCGCCCCGCTG
GTCGATCCGACGATGCGGCTGCTGGAGCGGGTGCTGCGCGACCCGAGCCGCGGCCGC
GCGGACGTGGACTGGTCGGAGGTGGCCGGCATCTATGTGGTCGGCGGGGCCGGCGGC

TTCCCGCTGGTGTCCCGGATGCTGCGCGCCCGGTTCGGGGACAAGCGGGTCAAGCGCT
CACCGCACCCGTTCGCCGCGACCGCCATCGGGTTGGCGGTGTTCTTGGACAAGGAATC
GGGTTTTGCGCTGTCCGAACGCTTTTCGCGGAATTTCGGGGTGTTCCGGGAGGCGGAG
GCGGGCGCCGGGGTGGTGTTCGACCCGATCGTGTGCAAGGACGTGTCGCTGCCGGCC
GACGGGCAGACCCCGCTGGTGGTCAAGCGGCGATACCGCGCCGCGCACAACATCGGG
CACTTCCGGTTCGTGGAGTGCAGCCGGCTGGTCGACGGGCGACCCGACGGCGACATC
ACCCCGTATGACCCGGTGCTGTTTCCGTTCGACCCGGCGCTGCGCGACCGCGACGACC
TGGGCCGCCAGCCGGTCGGCCGGTGGCGGGACGGACCCGACGTCGAGGAGCGCTACG
TGATCGCGCCCAGCGGTGCGGTGGAGGTGACGTTGACGACGCAGCCGGCCGGCTTCG
AACGCACCTTCCGGCTGGAGCGGTGCGCATCGGCGAGCCAAGCTTGCACCGCCGCCA
CCGACAGCTACAAGATGTCCGGCGGGCAGGGCTTCGCCATCCCGATCGGCCGCGCCAT
GGCCGTCGCCAACCAGATCCGCTCCGGCGCCGGCTCCAACACCGTGCACATCGGGCCC
ACCGCATTCCTCGGGCTGGGCGTGACGGACAACAACGGCAACGGCGCGCGGGTGCAG
CGGGTGGTCAACACCGGCCCGGCCGCGGCCGCGGGCATCGCGCCCGGCGACGTCATC
ACCGGCGTCGACACCGTCCCGATCAACGGGGCGACGTCGATGACCGAGGTGCTCGTC
CCGCACCACCCCGGTGACACCATCGCGGTGCACTTCCGGTCCGTCGACGGCGGCGAG
CGCACCGCGAACATCACCCTGGCGGAGGGGCCGCCGGCCACTCGAGCACCACCACCA
CCACCACTGAGATCCGGCTGCTAA

4-2. The amino acid sequences of 90NC (868aa)

MGSSHHHHHHSSGLVPRGSHMAPSGLALDRFADRPLAPIDPSAMVGQVGPQVVNIDTKF
GYNNAVGAGTGIVIDPNGVVLTNNHVISGATEISAFDVGNGQTYAVDVVGYDRTQDIAVL
QLRGAAGLPTATIGGEATVGEPIVALGNVGGQGGTPNAVAGKVVALNQSVSATDTLTGAQ
ENLGGLIQADAPIKPGDSGGPMVNSAGQVIGVDTAEFMRVGIDFGTTHTVVAAVDRGNYP
VVFFDGMDAWPSIIAANAAGELRYGADAAAVRHDPSWSVLRSFKRLLNDAGPQTRVKLA
GRDYRLAELLTGFLARLKTDLQQHSNATVAPGEPIEAAISVPANASSAQRLLTLDAYVAAG
FHVVALLNEPSAASLEYAHRYRSTITAKREYVLVYDLGGGTFDASLLKMTGHSNEVVVSE
GIQRLGGDDFDEAIVELVRAGADLPGLDAAGRALLAEECAARKEAVGPQTRRFLVDLSPF
DRPPFSCPVDDVYAACAPLVDPTMRLLERVLRDPSRGRADVDWSEVAGIYVVGGAGGFP
LVSRMLRARFGDKRVKRSPHPFAATAIGLAVFLDKESGFALSERFSRNFGVFREAEAGAGV
VFDPIVCKDVSLPADGQTPLVVKRRYRAAHNIGHFRFVECSRLVDGRPDGDITPYDPVLFP
FDPALRDRDDLGRQPVGRWRDGPDVEERYVIAPSGAVEVTLTTQPAGFERTFRLERCASAS
QACTAATDSYKMSGGQGFAIPIGRAMAVANQIRSGAGSNTVHIGPTAFLGLGVTDNNGNG
ARVQRVVNTGPAAAAGIAPGDVITGVDTVPINGATSMTEVLVPHHPGDTIAVHFRSVDGG
ERTANITLAEGPPATRAPPPPPLRSGC
