# Supplementary material for: A candidate subunit vaccine induces protective immunity against Mycobacterium avium subspecies paratuberculosis in mice
Source: NPJ Vaccines. 2023 May 20;8:72. doi: 10.1038/s41541-023-00675-1 (PMC10199288; doi:10.1038/s41541-023-00675-1)
Supplement: Supplementary file 2 — REPORTING SUMMARY [file 41541_2023_675_MOESM2_ESM.pdf]

nature portfolio

Corresponding author(s): NPJVACCINES-02855R1

Last updated by author(s): May 3, 2023

Reporting Summary

Nature Portfolio wishes to improve the reproducibility of the work that we publish. This form provides structure for consistency and transparency in reporting. For further information on Nature Portfolio policies, see our Editorial Policies and the Editorial Policy Checklist.

Statistics

For all statistical analyses, confirm that the following items are present in the figure legend, table legend, main text, or Methods section.

n/a	Confirmed	
☐	☒	The exact sample size (*n*) for each experimental group/condition, given as a discrete number and unit of measurement
☐	☒	A statement on whether measurements were taken from distinct samples or whether the same sample was measured repeatedly
☐	☒	The statistical test(s) used AND whether they are one- or two-sided<br>*Only common tests should be described solely by name; describe more complex techniques in the Methods section.*
☒	☐	A description of all covariates tested
☐	☒	A description of any assumptions or corrections, such as tests of normality and adjustment for multiple comparisons
☐	☒	A full description of the statistical parameters including central tendency (e.g. means) or other basic estimates (e.g. regression coefficient) AND variation (e.g. standard deviation) or associated estimates of uncertainty (e.g. confidence intervals)
☐	☒	For null hypothesis testing, the test statistic (e.g. *F*, *t*, *r*) with confidence intervals, effect sizes, degrees of freedom and *P* value noted<br>*Give P values as exact values whenever suitable.*
☒	☐	For Bayesian analysis, information on the choice of priors and Markov chain Monte Carlo settings
☒	☐	For hierarchical and complex designs, identification of the appropriate level for tests and full reporting of outcomes
☐	☒	Estimates of effect sizes (e.g. Cohen's *d*, Pearson's *r*), indicating how they were calculated

Our web collection on statistics for biologists contains articles on many of the points above.

Software and code

Policy information about availability of computer code

Data collection	no software was used
Data analysis	All statistical analyses were performed using GraphPad Prism 9.0.0 and R version 4.0.2

For manuscripts utilizing custom algorithms or software that are central to the research but not yet described in published literature, software must be made available to editors and reviewers. We strongly encourage code deposition in a community repository (e.g. GitHub). See the Nature Portfolio guidelines for submitting code & software for further information.

Data

Policy information about availability of data

All manuscripts must include a data availability statement. This statement should provide the following information, where applicable:

- Accession codes, unique identifiers, or web links for publicly available datasets
- A description of any restrictions on data availability
- For clinical datasets or third party data, please ensure that the statement adheres to our policy

We have provided a full data availability statement in the manuscript.

Human research participants

Policy information about studies involving human research participants and Sex and Gender in Research.

Reporting on sex and gender	The study did not involve human research participants.
Population characteristics	The study did not involve human research participants.
Recruitment	The study did not involve human research participants.
Ethics oversight	The study did not involve human research participants.

Note that full information on the approval of the study protocol must also be provided in the manuscript.

Field-specific reporting

Please select the one below that is the best fit for your research. If you are not sure, read the appropriate sections before making your selection.

☒ Life sciences ☐ Behavioural & social sciences ☐ Ecological, evolutionary & environmental sciences

For a reference copy of the document with all sections, see nature.com/documents/nr-reporting-summary-flat.pdf

Life sciences study design

All studies must disclose on these points even when the disclosure is negative.

Sample size	Data indicate cumulative results from at least three to six independent replicates. One-way and two-way ANOVA were used to analyze statistical significance, followed by Tukey's multiple comparisons tests.
Data exclusions	No data were excluded from the analyses.
Replication	All attempts at replication were successful.
Randomization	The mice were randomly allocated into experimental findings.
Blinding	Two independent pathologists quantified lung and intestine injury scoring in a double-blind manner in the study.

Reporting for specific materials, systems and methods

We require information from authors about some types of materials, experimental systems and methods used in many studies. Here, indicate whether each material, system or method listed is relevant to your study. If you are not sure if a list item applies to your research, read the appropriate section before selecting a response.

Materials & experimental systems

n/a	Involved in the study	
☐	☒	Antibodies
☒	☐	Eukaryotic cell lines
☒	☐	Palaeontology and archaeology
☐	☒	Animals and other organisms
☒	☐	Clinical data
☒	☐	Dual use research of concern

Methods

n/a	Involved in the study	
☒	☐	ChIP-seq
☐	☒	Flow cytometry
☒	☐	MRI-based neuroimaging

Antibodies

Antibodies used

1. HRP conjugated Rabbit Anti-mouse IgG H&L, Supplier name: Abcam, Catalog numer: ab6728, Clone name: no, Lot number: GR3289920-6, Application: Dot blot, ELISA, IHC-P, IHC-Fr, Immunomicroscopy, ICC/IF.
2. HRP conjugated Goat Anti-mouse IgM mu chain, Supplier name: Abcam, Catalog numer: ab97230, Clone name: no, Lot number: GR3336726-4, Application: ELISA, WB, ICC, IHC;
3. HRP conjugated Goat Anti-mouse IgG1, Supplier name: Abcam, Catalog numer: ab97240, Clone name: no, Lot number: GR3320187-8, Application: ICC, ELISA, IHC-P, WB.
4. HRP conjugated Goat Anti-mouse IgG2a heavy chain, Supplier name: Abcam, Catalog numer: ab97245, Clone name: no, Lot

number: GR3324477-7, Application: IHC-P, ELISA, WB, ICC.
5. PE-conjugated CD4 Monoclonal Antibody, Supplier name: ThermoFisher, Catalog numer: 12-0041-82, Clone name: GK1.5, lot number: 2376147, Application: WB, IHC, ICC/IF, Flow, FN. Species: Fish, Human, Mouse
6. PE-conjugated CD8 alpha Monoclonal AntibodySupplier name: ThermoFisher, Catalog numer: MCD0804, Clone name: 5H10, Lot number: 2170194, Application: Flow. Species: Mouse.
7. PE/Cy5-conjugated Anti-IFN-γ, Supplier name: Abcam, Catalog numer: ab272255, Clone name: XMG1.2, Lot number: GR3394901-3, Application: Flow. Species: Mouse.
8. Alexa Fluor 647-conjugated Rat anti-Mouse IL-17A, Supplier name: BD Biosciences, Catalog numer: 560184, Clone name: TC11-18H10, Lot number: 9352425, Application: Intracellular staining (flow cytometry). Species: Mouse.
9. eFluor 450-conjugated anti-Mouse TNF-α, Supplier name: ThermoFisher, Catalog numer: 48-7321-82, Clone name: MP6-XT22, Lot number: 2373837, Application: Flow, IHC, Neu. Species: Mouse.
10. PE/Cy5-conjugated Rat IgG1 Kappa Light Chain Isotype, Supplier name: NOVUS Biologicals, Catalog numer: NBP1-43076, Clone name: RG1, Lot number: 2082873, Application: Flow-Isotype Control.
11. Alexa Fluor 647-conjugated Rat IgG1, Supplier name: SouthernBiotech, Catalog numer: 0116-31, Clone name: KLH/G1-2-2, Lot number: K1312-P9748, Application: Flow Cytometry, ELISA , FLISA , Immunocytochemistry, Blocking, In vitro Control, In vivo Control.
12. eFluor 450-conjugated Rat IgG1 kappa Isotype Control Supplier name: ThermoFisher, Catalog numer: 48-4301-82, Clone name: eBRG1, Lot number: 2082873, Application: Flow-Isotype Control.

Validation

1. PE-conjugated CD4 Monoclonal Antibody, Application: WB, IHC, ICC/IF, Flow, FN. Species: Fish, Human, Mouse
2. PE-conjugated CD8 alpha Monoclonal Antibody, Application: Flow. Species: Mouse.
3. PE/Cy5-conjugated Anti-IFN-γ, Application: Flow. Species: Mouse.
4. Alexa Fluor 647-conjugated Rat anti-Mouse IL-17A, Application: Intracellular staining (flow cytometry). Species: Mouse.
5. eFluor 450-conjugated anti-Mouse TNF-α, Application: Flow, IHC, Neu. Species: Mouse.

Animals and other research organisms

Policy information about studies involving animals; ARRIVE guidelines recommended for reporting animal research, and Sex and Gender in Research

Laboratory animals	Six-week-old female C57BL/6 mice.
Wild animals	The study did not involve wild animals.
Reporting on sex	The findings apply to only one sex, and the sex was not considered in the study.
Field-collected samples	The study did not involve samples collected from the field.
Ethics oversight	This study was approved by the Animal Ethics committee of Harbin Veterinary Research Institute, China (Ethical Committee Approval number HVRI-IACUC-200723-01).

Note that full information on the approval of the study protocol must also be provided in the manuscript.

Flow Cytometry

Plots

Confirm that:

☒ The axis labels state the marker and fluorochrome used (e.g. CD4-FITC).

☐ The axis scales are clearly visible. Include numbers along axes only for bottom left plot of group (a 'group' is an analysis of identical markers).

☒ All plots are contour plots with outliers or pseudocolor plots.

☒ A numerical value for number of cells or percentage (with statistics) is provided.

Methodology

Sample preparation	The lymphocytes were isolated from the spleen of mice using the Mouse Spleen Lymphocyte Separation Medium Kit (TBD Science, Tianjin, China) according to the manufacturer's instructions. The details of the procedure are as follows. The mouse spleen was aseptically separated, cut into small pieces, and ground in the homogenate rinse fluid (TBD Science). The single-cell suspension was obtained through a 70-mesh screen, and then centrifuged at 350 × g for 10 min and the supernatants were discarded. All tissue cells were resuspended in tissue sample diluent (TBD Science) and then added onto the surface of lymphocyte separation medium (TBD Science). The spleen lymphocytes were obtained by centrifugation at 400 × g for 30 min and then washed with a wash solution (TBD Science).
Instrument	Apogee A50-Micro Nanoscale Flow Cytometry
Software	Apogee Histogram software, PE-labeled CD4 and CD8 cells were detected in the Org channel (488 nm). PE/Cy5-labeled cells secreting IFN-γ were detected in the red channel (488 nm). Alexa Fluor 647-labeled cells secreting IL-17A cells were detected in the red channel (638 nm). eFluor 450-labeled cells secreting TNF-α cells were detected in the Blu channel (405 nm).

Cell population abundance	The lymphocytes were isolated from the spleen of mice using the Mouse Spleen Lymphocyte Separation Medium Kit (TBD Science, Tianjin, China). The lymphocytes were purified by removing the adhering cells.
Gating strategy	The gating strategies were as follows: CD4+ or CD8+ T cells was the first gated and then other markers were detected within the gates. CD4-positive or CD8-positive T cells were defined as positive T cell population and CD4-negative or CD8-negative T cells were defined as negative T cell population.

☒ Tick this box to confirm that a figure exemplifying the gating strategy is provided in the Supplementary Information.
